# SGLT2 inhibition protects kidney function by SAM-dependent epigenetic repression of inflammatory genes under metabolic stress

**DOI:** 10.1172/JCI188933

**Published:** 2025-10-01

**Authors:** Hiroshi Maekawa, Yalu Zhou, Yuki Aoi, Margaret E. Fain, Dorian S. Kaminski, Hyewon Kong, Zachary L. Sebo, Ram P. Chakrabarty, Benjamin C. Howard, Grant Andersen, Biliana Marcheva, Peng Gao, Pinelopi Kapitsinou, Joseph Bass, Ali Shilatifard, Navdeep S. Chandel, Susan E. Quaggin

**Affiliations:** 1 Feinberg Cardiovascular & Renal Research Institute,; 2 Division of Nephrology & Hypertension, Department of Medicine,; 3 Department of Biochemistry and Molecular Genetics, and; 4 Simpson Querrey Institute for Epigenetics, Feinberg School of Medicine, Northwestern University, Chicago, Illinois, USA.; 5 Department of Medicine & Biochemistry and Molecular Genetics, Northwestern University, Chicago, Illinois, USA.; 6 Department of Medicine, Division of Endocrinology, Metabolism, and Molecular Medicine, and; 7 Robert H. Lurie Cancer Center Metabolomics Core, Feinberg School of Medicine, Northwestern University, Chicago, Illinois, USA.

**Keywords:** Metabolism, Nephrology, Diabetes, Epigenetics, NF-kappaB

## Abstract

Clinically, blockade of renal glucose resorption by sodium–glucose cotransporter 2 (SGLT2) inhibitors slows progression of kidney disease, yet the underlying mechanisms are not fully understood. We hypothesized that altered renal metabolites underlie observed kidney protection when SGLT2 function is lost. *S*-adenosylmethionine (SAM) levels were increased in kidneys from mice lacking SGLT2 function on a diabetogenic high-fat diet (SP^HFD^) compared with WT mice fed HFD. Elevated SAM in SP^HFD^ was associated with improved kidney function and decreased expression of NF-κB pathway–related genes. Injured proximal tubular cells that emerged under HFD conditions in WT mice and humans consistently showed reduction in expression of the SAM synthetase *Mat2a/MAT2A*, while MAT2A inhibition, which reduces SAM production, abrogated kidney protection in SP^HFD^ mice. Histone H3 lysine 27 (H3K27) repressive trimethylation of NF-κB–related genes was increased in SP^HFD^, consistent with SAM’s role as a methyl donor. Our data support a model whereby SGLT2 loss enhances SAM levels within the kidney, leading to epigenetic repression of inflammatory genes and kidney protection under metabolic stress.

## Abstract 2

SGLT2 inhibition protects kidneys from metabolic injury through increased tissue levels of the methyl donor S-adenosyl methionine (SAM), resulting in enhanced H3K27 trimethylation and repression of inflammatory genes.

## Graphical abstract

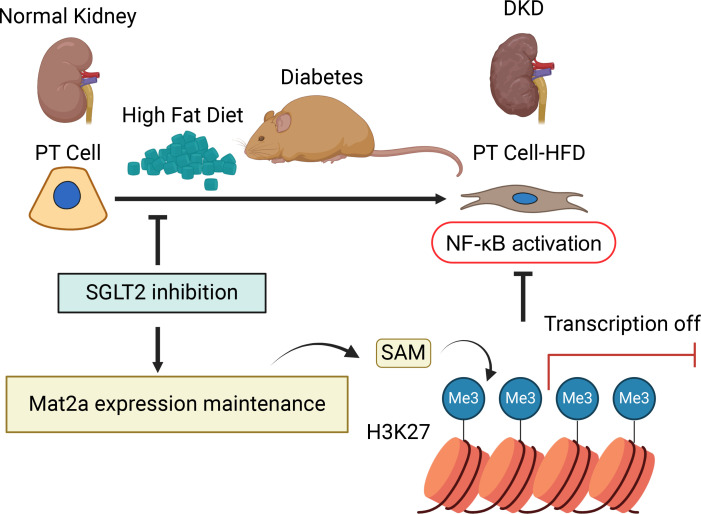

## Introduction

Diabetic kidney disease (DKD) is a leading cause of chronic kidney disease (CKD) and end stage kidney failure worldwide. DKD develops in approximately 40% of patients with diabetes (1). In 2022, an estimated 828 million adults had diabetes, and the number continues to rise (2). A relatively new class of therapeutic agents, sodium–glucose cotransporter 2 (SGLT2) inhibitors, which were originally developed to improve glucose control by promoting glucosuria, have demonstrated powerful kidney and cardiovascular benefits and reduced mortality in patients with diabetes (3–5). More recently, multiple clinical trials showed renoprotective effects in CKD patients with and without diabetes, suggesting SGLT2 inhibitors have effects beyond glucose control (6, 7). A peculiar aspect of SGLT2 is the restricted expression only in the proximal convoluted tubule in the kidney (8, 9). It is not well understood how targeting this transporter in a small subset of cells improves overall kidney function. In kidneys from diabetic and nondiabetic patients and rodents with CKD, several metabolic alterations, including increased glycolysis, TCA cycle, and fatty acid metabolism as well as mitochondrial dysfunction, have been reported (10–13). SGLT2 inhibition normalized these metabolic alterations in rodent models, associated with renal protection (14, 15). Furthermore, SGLT2 inhibitors stimulated ketogenesis and lipolysis as a physiological adaptive response against the continuous glucose loss (16–18). Ketone body production has been reported to protect kidneys in diabetes via inhibition of mTORC1 activation (18). Despite these important studies, the contributions of intrinsic metabolic effects of SGLT2 inhibition to the renoprotective effect have not been fully elucidated (9, 19). Given that SGLT2 inhibitors function by blocking proximal tubular cell (PTC) uptake of glucose, it is reasonable to posit there will be important metabolic changes within kidney cells themselves. Since metabolites have been shown to control cellular function and fate in different contexts (20, 21), here we tested the hypothesis that metabolites and metabolic pathways are altered in PTCs that have lost SGLT2 function and that these pathways are kidney protective. Furthermore, we propose that these pathways will provide new candidate therapeutic targets for patients who are unable to tolerate SGLT2 inhibitors and identify previously unknown targets that might be more powerful or work in concert with SGLT2 inhibitors to enhance overall treatment benefit.

Here, we used metabolomic, transcriptomic, and epigenetic approaches to test our hypothesis and to explore early changes in kidney cells that promote kidney protection. These studies revealed a biologic role for methionine metabolism and the *S*-adenosylmethionine (SAM) metabolite as being necessary for epigenetic repression of inflammatory gene pathways that provide kidney protection in the setting of a high-fat diet (HFD).

## Results

### Kidneys of mice lacking SGLT2 function are protected from injury in HFD conditions.

**Figure 1:**
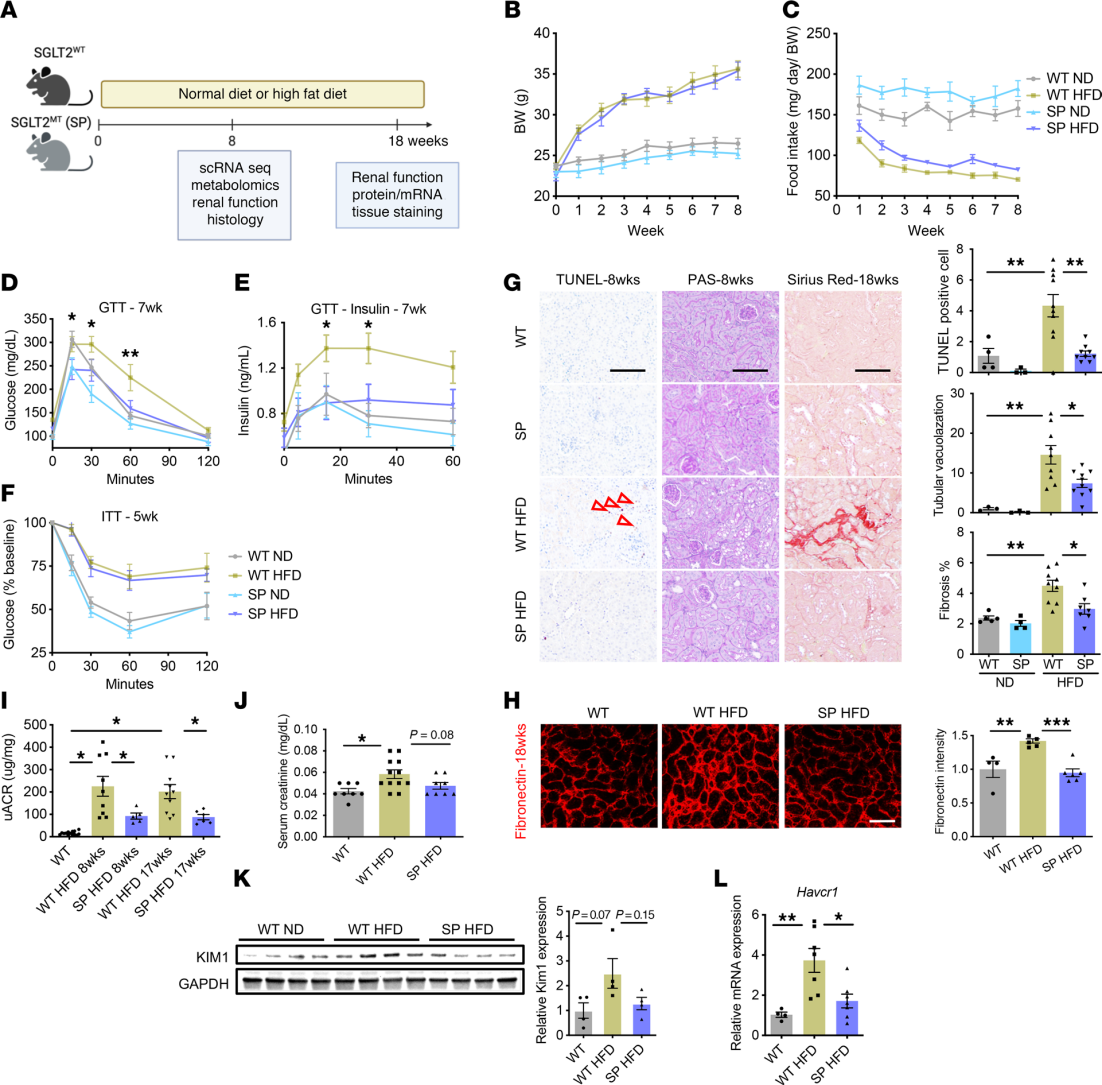
LOF of SGLT2 improved glucose intolerance and kidney injury. (**A**) Schematic of experimental protocol. (**B**) Chronological BW change. (**C**) Chronological changes in food intake. (**D**–**F**) HFD elevated glucose intolerance (**D** and **E**) and insulin resistance (**F**). Glucose intolerance was blunted in SP mice (**D** and **E**). GTT, glucose tolerance test; ITT, insulin tolerance test. (**G**) Representative images of TUNEL, PAS, and Sirius Red staining at 8 or 18 week feeding time points across groups. The arrows indicate TUNEL-positive cells. Right panel, quantification. (**H**) Representative images of fibronectin immunofluorescence in the kidney cortex at the 18 week feeding time point. Right panel, quantification. (**I**) uACR at 8 and 17 week feeding time points. (**J**) Serum creatinine level. (**K**) Protein level of KIM1 in renal cortex of mice at the 18 week feeding time point. Right panel, quantification. (**L**) RT-qPCR analysis of Havcr1. Scale bars: 100 μm. Sample numbers: WT-ND 8wks, *n* = 10; SP-ND 8wks, *n* = 9; WT-HFD 8wks, *n* = 14; SP-HFD 8wks, *n* = 13; WT-ND 18wks, *n* = 7; WT-HFD 18wks, *n* = 12; SP-ND 18wks, *n* = 4; SP-HFD 18wks, *n* = 8. Data were analyzed by 1-way (**G**–**L**) or 2-way (**B**–**F**) ANOVA; ***P < 0.001, **P < 0.01, and *P < 0.05 by Tukey’s test. Values are presented as mean ± SEM.

We used a mouse model known as Sweet Pee (SP) that carries a missense mutation in the *Slc5a2* gene encoding the SGLT2 cotransporter. Homozygous mice carrying 2 mutant alleles did not make functional SGLT2 protein and exhibit glucosuria (Supplemental Figure 1A; supplemental material available online with this article; https://doi.org/10.1172/JCI188933DS1) (22). To induce metabolic syndrome, 10-week-old SP or WT mice were fed a HFD (60% calories from fat) for 8 or 18 weeks (Figure 1A). Mice of both genotypes showed similar weight gain at both time points, while compensatory hyperphagia was observed in SP mice (Figure 1, B and C, and Supplemental Figure 1B). HFD led to elevation of postprandial blood glucose level, glucose intolerance, and insulin resistance (Figure 1, D–F, and Supplemental Figure 1C). Increases in postprandial blood glucose and glucose intolerance were present but blunted in SP^HFD^ mice that displayed glucosuria (Figure 1, D and E, and Supplemental Figure 1C). As early as 8 weeks of HFD, TUNEL staining was increased, and periodic acid–Schiff (PAS) staining demonstrated more tubular vacuolization in kidneys from WT^HFD^ versus SP^HFD^ mice. By 18 weeks, Sirius Red and fibronectin staining showed a mild increase in tubulointerstitial fibrosis, and early changes in mesangial expansion in WT^HFD^ mice were observed (Figure 1, G and H, and Supplemental Figure 1D) compared with SP^HFD^ mice. Kidney function tests demonstrated more injury in WT mice fed HFD compared with SP^HFD^ mice, as estimated by serum creatinine and urine albumin/creatinine ratio (uACR) (Figure 1, I and J). After 18 weeks of HFD, KIM-1, a marker of renal PTC injury, and markers of fibrosis (fibronectin, αSMA, and SM22 α) and apoptosis (cleaved caspase-3) were upregulated in WT mice and abrogated in SP mice (Figure 1, K and L, and Supplemental Figure 1E). Additional evidence of activation of fibrotic gene pathways was confirmed by increased expression of fibrotic genes (*Tagln*, *Acta2*, *Fn1*, *Vim*, *Col1a1*, and *Col3a1*) in the renal cortex of WT^HFD^ compared with SP^HFD^ (Supplemental Figure 1F). Interestingly, although HFD led to increased kidney weight in both WT and SP mice, it was greater in SP > WT mice (Supplemental Figure 1G), consistent with the observed decline in kidney function in WT mice. This size difference between genotypes was not observed in the heart (Supplemental Figure 1H). Taken together, genetic loss of function (LOF) of SGLT2 and reduction in glucose transport in renal PTCs ameliorated renal injury in a model of early diabetes secondary to HFD.

### Emergence of a population of PTCs with injury phenotype in the setting of HFD.

**Figure 2:**
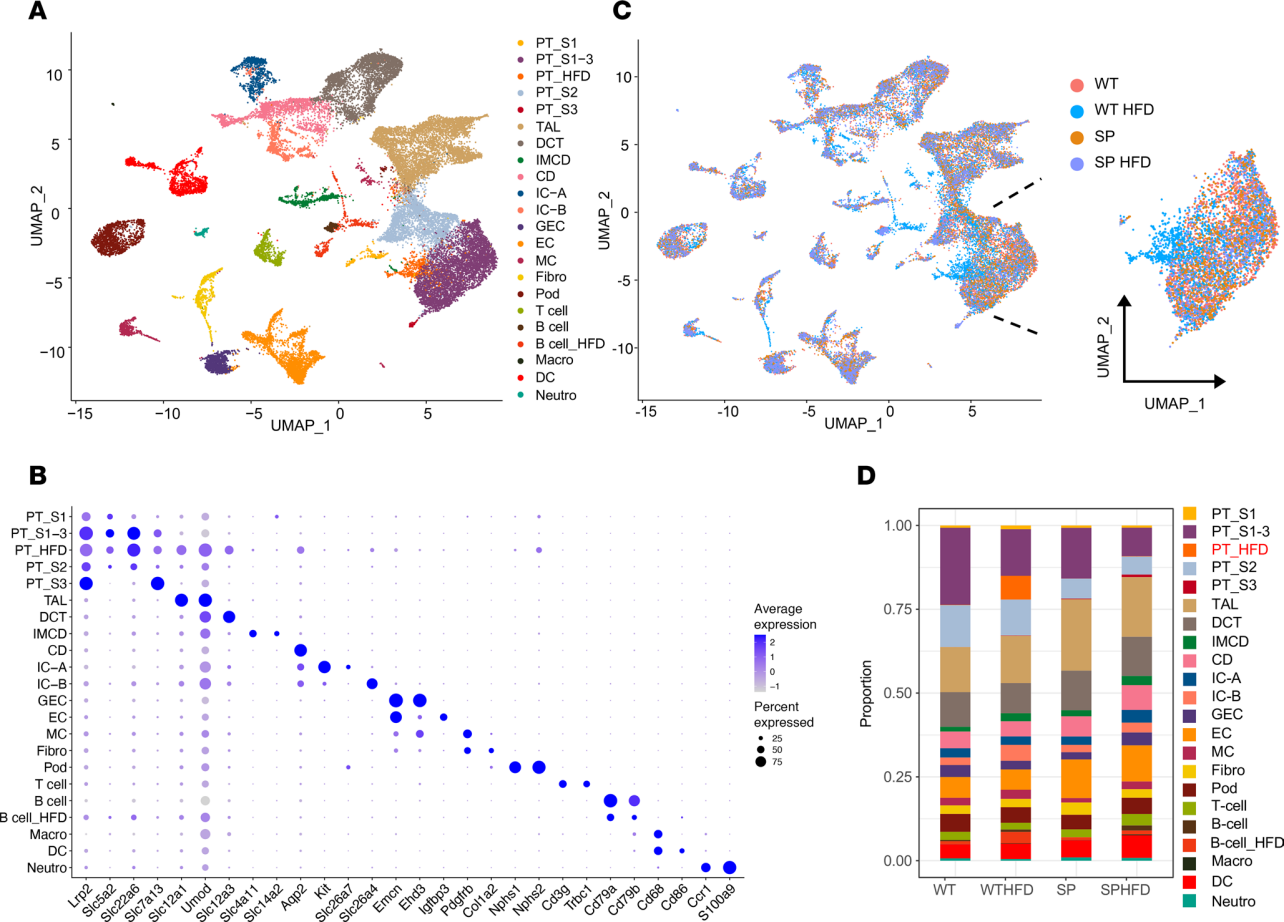
Population of injured PTCs is enriched in WT^HFD^ mice. (**A**) Uniform Manifold Approximation and Projection (UMAP) demonstrating 22 distinct cell types in kidney. (**B**) Dot plot of canonical cell marker genes (size of the dot indicates the percent positive cells, and color indicates relative expression). (**C**) UMAP colored by experimental groups. Right panel shows PTC clusters by genotype. (**D**) Stacked bar plot displaying distribution of relative cell percentage of total cells. PT, proximal tubule; TAL, thick ascending limb of the loop of Henle; DCT, distal convoluted tubule; IMCD, inner medullary collecting duct; CD, collecting duct; IC, intercalated cells; GEC, glomerular endothelial cell; EC, endothelial cell; MC, mesangial cell; Pod, podocyte; DC, dendritic cell.

To determine early transcriptional changes in kidney cells triggered by HFD, single-cell RNA-Seq (scRNA-Seq) was performed as described (23). We analyzed 30,958 single cells isolated from whole kidneys of WT or SP mice with/without HFD feeding. Unbiased clustering identified 30 cell clusters, including renal epithelial, immune, endothelial, and interstitial cells (Supplemental Figure 2A). Based on canonical marker gene expression signatures, annotation was performed (Figure 2, A and B). A new subcluster of PTCs (hereafter referred to as PT-HFD) was detected in the kidney following HFD (Figure 2, A and B), and these cells were predominantly observed in kidneys from WT^HFD^ mice compared with normal diet (ND) groups and SP^HFD^ mice (Figure 2, C and D). We confirmed that the percentage of ambient RNA remains consistently low across all 4 samples by approximately 5% (Supplemental Figure 3A). Additionally, we assessed mitochondrial RNA levels, total RNA counts, and feature counts per cluster. The number of total RNA detected features and the proportion of mitochondrial RNA in PT-HFD were comparable with those observed in other cell clusters (Supplemental Figure 3B). PT-HFD cells expressed canonical PTC genes, including *Lrp2*, *Slc5a2*, *Slc22a6*, and *Slc7a13*, but they also expressed *Slc12a1* and *Umod*, markers of the thick ascending limb (Figure 2B and Supplemental Figure 2B). We confirmed that *Slc12a1* and *Umod* are expressed in injured PTCs identified in another published human kidney RNA-Seq dataset (data not shown) (24). Of note, expression of *Slc5a1*, the gene encoding SGLT1, was unchanged among all groups, demonstrating no compensatory upregulation of this cotransporter in PTCs following loss of SGLT2 (Supplemental Figure 4, A and B).

### RNA-Seq analysis identified metabolic and inflammatory gene signatures in PT-HFD.

**Figure 3:**
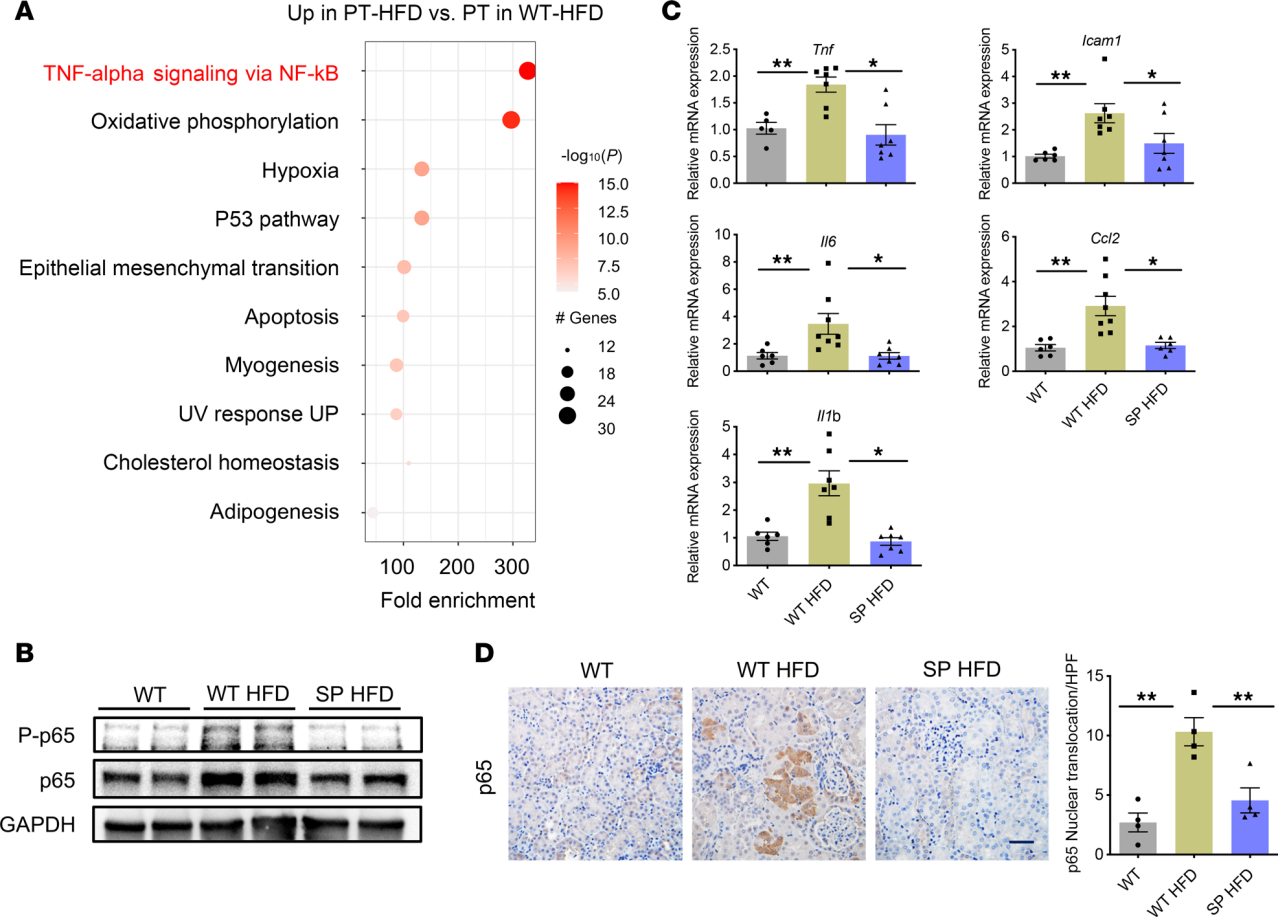
Inflammatory gene pathways are enriched in the PT-HFD cluster. (**A**) Bubble plot showing pathway enrichment of upregulated pathways in PT-HFD (size of the dot indicates the percent positive cells, and color indicates relative expression). (**B**) Western blot analysis of phosphorylated/total p65 in tissue isolated from the renal cortex across groups at the 18 week feeding time point. (**C**) RT-qPCR analysis of proinflammatory cytokines and chemokines in tissue isolated from the renal cortex across groups (*n* = 6–8 per group). (**D**) Representative images of immunohistochemistry of p65 in the kidney and quantification (right panel). Scale bar: 100 μm. Data were analyzed by 1-way ANOVA; ***P < 0.001, **P < 0.01, and *P < 0.05 by Tukey’s test. Values are presented as mean ± SEM.

Because of the known alteration of metabolic gene signatures in PTCs in the setting of diabetes, we explored changes in metabolism-related gene signatures in PT-HFD. Consistent with prior studies (12–14), we observed decreased gene expression related to fatty acid oxidation (e.g., *Ppara* and *Acadm*) (Supplemental Figure 5A) and gluconeogenesis-related genes (e.g., *G6pc* and *Hnf4a*), while genes encoding glycolytic enzymes (e.g., *Hk1* and *Pkm*) were upregulated in PT-HFD (Supplemental Figure 5B). Pathway enrichment analysis of the top ranked differentially expressed genes for cells in the PT-HFD cluster was performed using the Hallmark gene set (Supplemental Table 1). The most upregulated pathway in the PT-HFD was related to genes in the TNF-α signaling via NF-κB pathway (Figure 3A). Moreover, several NF-κB target genes, including *Il1*β, were upregulated in total PTCs of WT^HFD^ but were not increased in PTCs of SP^HFD^ (Supplemental Figure 5C).

### Validation of genes related to inflammation.

Given the prominent role that inflammation is known to play in progression of kidney disease in the setting of diabetes as well as in other kidney diseases that respond favorably to SGLT2 inhibitors (18, 25, 26), we performed additional studies to validate transcriptomic changes related to the NF-κB pathway.

In keeping with increased NF-kB activity, Western blot analysis showed the expression of phospho-/total NF-kB p65 was increased in renal cortex harvested from WT^HFD^ mice compared with SP^HFD^ mice (Figure 3B). Similarly, reverse transcription quantitative PCR (RT-qPCR) of renal cortex tissue showed increased expression of inflammatory cytokines (*Tnf*, *Il1*β, *Ccl2*, *Il6*, and *Icam1*) in WT^HFD^ versus SP^HFD^ mice (Figure 3C), consistent with the increased proportion of PT-HFD identified in RNA-Seq datasets from WT^HFD^ mice (Figure 2D). Immunohistochemistry confirmed that nuclear translocation of NF-kB p65 was increased in PTCs in kidneys from WT^HFD^ compared with SP^HFD^ mice (Figure 3D). To determine whether the gene signature pathways identified in PT-HFD are relevant for humans and similar to those previously identified in other rodent models of diabetes (i.e., db/db), we interrogated and reanalyzed publicly available RNA-Seq datasets (26, 27). In particular, we were interested to see if a similar PT-HFD subcluster exists in human kidneys at early stages of diabetes. In humans with diabetes, we identified a subset of PTCs, termed PTC-D1, whose gene expression signature also resembled PT-HFD (Supplemental Figure 6, A–D). We also examined DKD samples in the KPMP dataset and found 2 distinct types of abnormal PTCs: adaptive and degenerative. Both cell types showed enrichment of NF-kB signaling pathways, which is consistent with our findings in mice (Supplemental Figure 7, A and B). Similarly, in a db/db model of diabetes in mice, we identified a cluster of injured PTCs, whose gene expression resembled that of PT-HFD (Supplemental Figure 8, A–D).

The Activator Protein-1 (AP-1) family of transcription factors, including FOS, JUN, and JUNB, cooperate with the NF-κB complex to promote inflammatory signals (28–31). To investigate whether expression levels of AP-1 family members also change in PTCs from HFD-fed mice and patients with early diabetes, we reanalyzed the scRNA-Seq/single-nucleus RNA-Seq kidney datasets. The expression levels of *Fos*, *Jun*, and *Junb* were increased in injured PTCs of HFD-fed mice and diabetic patients, i.e., PT-HFD and PTC-D1, respectively (Supplemental Figure 9, A and B). RNAscope analysis confirmed *Fos* upregulation in PTCs of WT^HFD^ (Supplemental Figure 9C). Moreover, the upregulation of *Fos* and *Jun* in the renal cortex did not occur in SP^HFD^ mice (Supplemental Figure 9D). Overall, genetic LOF of SGLT2 in mice was associated with suppression of NF-κB activation in PTCs compared with PTCs from WT mice under HFD/metabolic syndrome conditions.

### Methionine metabolism is altered in kidneys of mice under HFD conditions.

**Figure 4:**
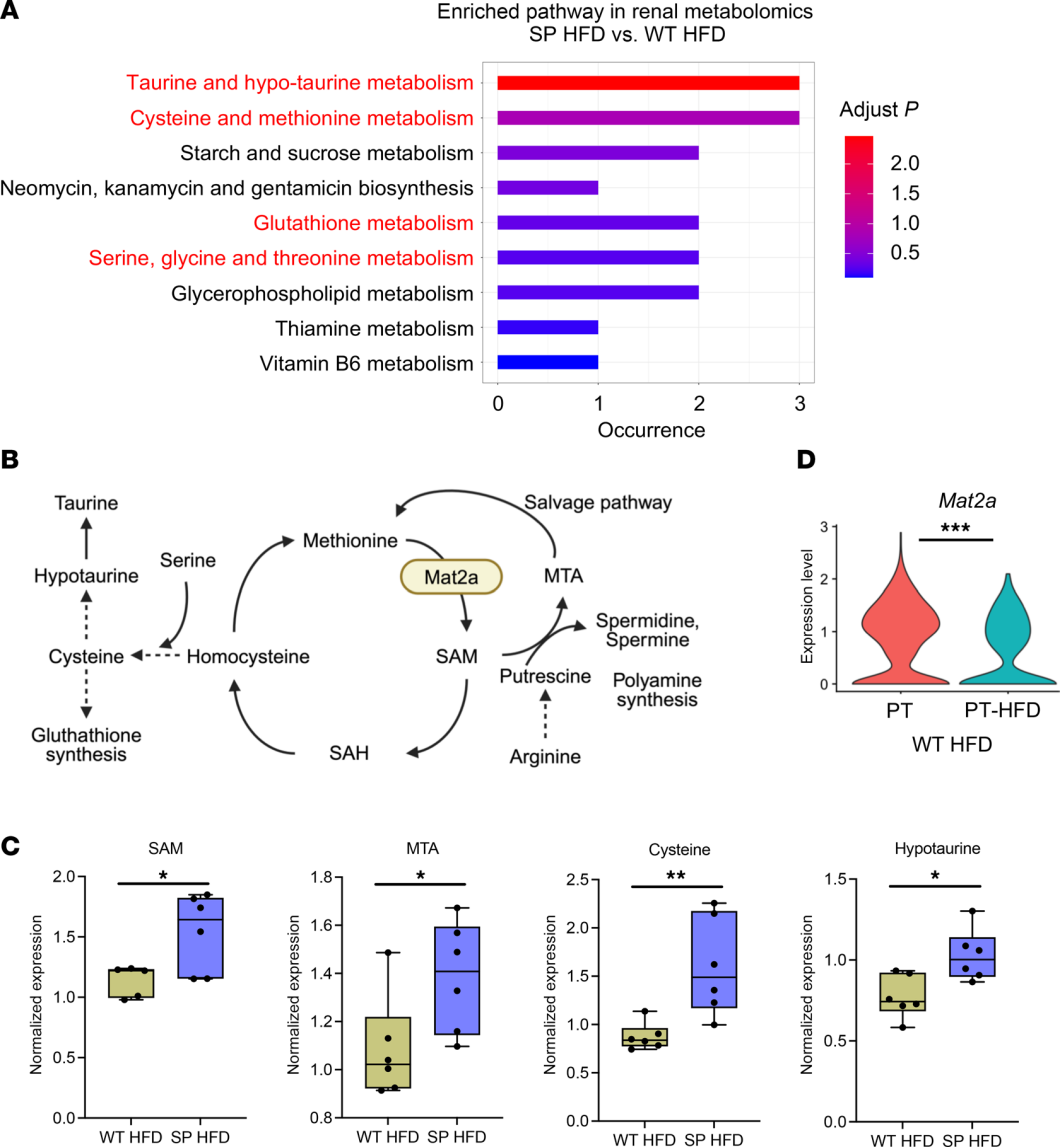
Metabolic profiles in the kidney. (**A**) Metabolic enrichment pathway analysis in tissue isolated from the renal cortex. Color indicates adjusted *P* value. (**B**) Methionine metabolism and network pathways. (**C**) Relative expression of SAM, MTA, and metabolites related to cysteine and taurine metabolism in renal cortex (*n* = 6 per group). Boxes show the 25th to 75th percentiles, center lines indicate medians, whiskers extend to min and max, and all data points are shown. **P < 0.01 and *P < 0.05; Student’s *t* test. (**D**) Violin plot showing *Mat2a* expression in PTC versus PT-HFD of the WT-HFD. ***Adjusted *P* < 0.001. Values are presented as mean ± SEM.

As our RNA-Seq analysis suggested changes in metabolic gene signatures when SGLT2 function is lost, metabolomic analysis was performed on tissue harvested from the renal cortex and serum of SGLT2 WT and SP mice under both diet conditions at 8 weeks. In the renal cortex, 57% of cellular diversity and 77% of protein mass are derived from PTCs (32), suggesting that the majority of metabolites in the renal cortex are derived from PTCs, the cell type that expresses the SGLT2 cotransporter. At the 8-week early time point before diabetes has been present for a prolonged period, as expected, most differences were driven by dietary differences rather than genotype differences. However, there were significant differences in the renal cortex and serum between genotypes (SP and WT) under HFD (Supplemental Figures 10 and 11 and Supplemental Tables 2 and 3). While pathway enrichment analysis identified several metabolic pathways that were upregulated in the renal cortex from SP^HFD^ mice compared with WT^HFD^ mice (Figure 4A), methionine metabolism (21, 33) is central to all of them (Figure 4B). Indeed, multiple metabolites within and/or impacted by the methionine pathway, including SAM, methylthioadenosine (MTA), cysteine, and hypotaurine, were increased in the renal cortex of SP^HFD^ mice (Figure 4C). Furthermore, expression of *Mat2a*, which is a SAM synthetase and catalyzes the formation of SAM from methionine, was significantly downregulated in PT-HFD, injured PTCs, and PTC-D1, the population of injured PTCs. A similar downward trend was observed in adaptive and degenerative PTC clusters in the KPMP dataset (Figure 4D, Supplemental Figure 6D, Supplemental Figure 7C, and Supplemental Figure 8C). The downregulation of *Mat2a* in injured PTCs compared with other PTC subclusters supports a model whereby dysregulation of methionine metabolism and subsequent reduction in SAM production in PTCs in the setting of HFD/metabolic syndrome are associated with the emergence of injured cells. Together, comprehensive metabolomic analysis detected methionine metabolic modulation as a target of SGLT2 inhibition within PTCs.

### Inhibition of the SAM synthetase MAT2A in SP^HFD^ mice abrogated kidney protection.

**Figure 5:**
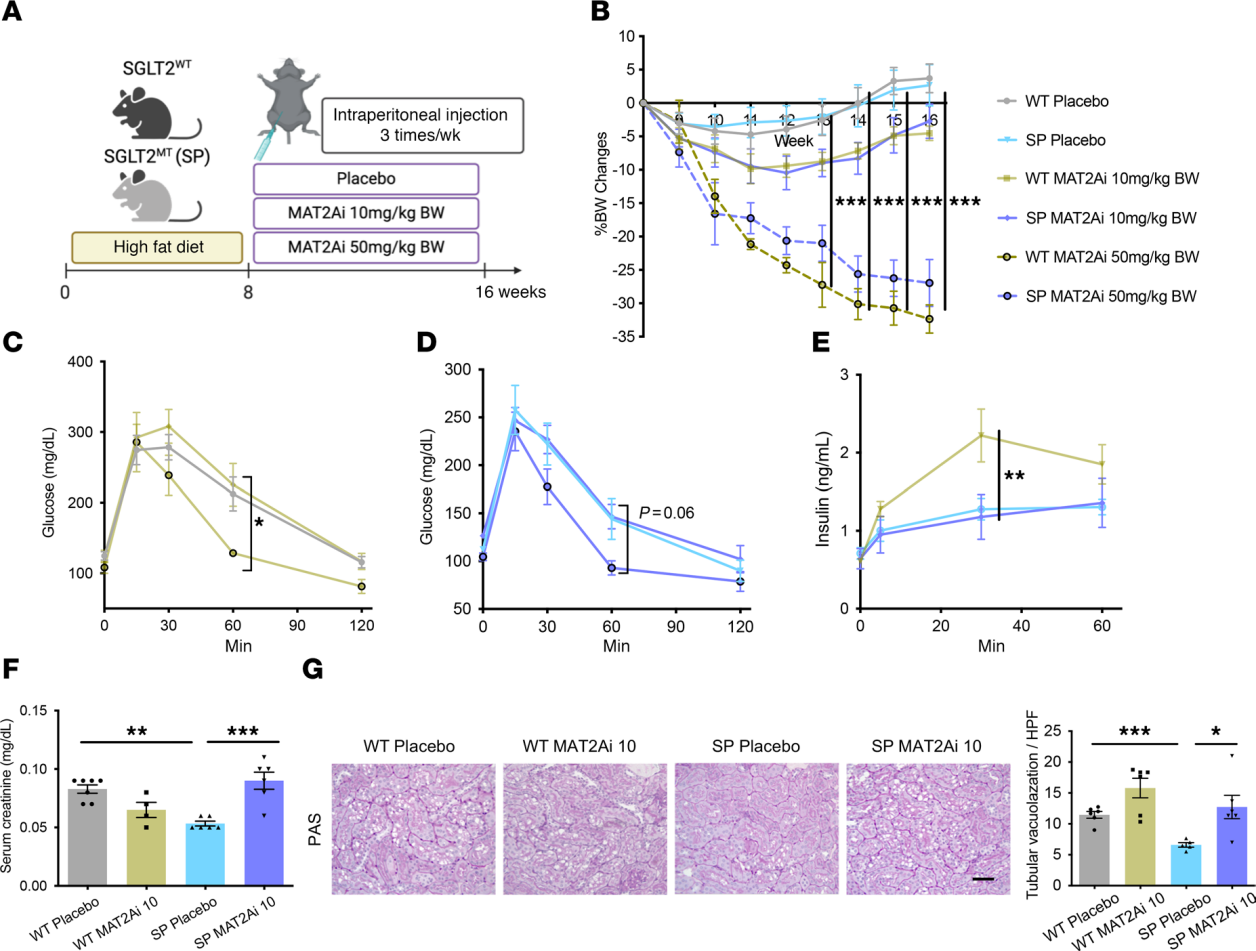
Inhibition of methionine enzyme, MAT2A, abrogates kidney protection in SP^HFD^ mice. (**A**) Schematic protocol. (**B**) Chronological changes in percentage of BW change. (**C**–**E**) Low-dose MAT2Ai does not alter glucose tolerance and insulin secretion capacity, but high dose MAT2Ai does lower them. (**F**) Serum creatinine level. (**G**) Representative images of PAS staining. Right panel, quantification. HPF, High-power field (original magnification ×40). Sample numbers: WT-PL, *n* = 7; WT-MAT2Ai LD, *n* = 7; WT-MAT2Ai HD, *n* = 3; SP-PL, *n* = 8; SP-MAT2Ai LD, *n* = 8; SP-MAT2Ai HD, *n* = 5. Scale bar: 50 μm. Data were analyzed by 1-way (**C**–**G**) or 2-way (**B**) ANOVA; ***P < 0.001, **P < 0.01, and *P < 0.05 by Tukey’s test. Values are presented as mean ± SEM.

Since SAM and *Mat2a* were both increased in the renal cortex and PTCs, respectively, of SP^HFD^ mice compared with WT^HFD^ mice (Figure 4, C and D), we hypothesized that SGLT2 inhibition protects the kidney by revving up methionine metabolism and SAM levels within PTCs. To test this hypothesis and determine whether MAT2A is required to protect PTCs in the setting of HFD, WT^HFD^ and SP^HFD^ mice were treated with MAT2A inhibitor (MAT2Ai) to block production of SAM. As expected, SAM levels were lower (trend) in kidneys from SP mice treated with a MAT2Ai compared with vehicle (Supplemental Figure 12). Two dosages of MAT2Ai (10 [low dosage, LD] or 50 [high dosage, HD] mg/kg BW) were tested. After 8 weeks of preconditioning with HFD, WT or SP mice were assigned to i.p. injection of placebo, LD, or HD MAT2Ai 3 times a week for 8 weeks (Figure 5A). The HD led to severe weight loss in both genotypes consistent with general toxicity of the inhibitor (Figure 5B). Conversely, BW reduction, glucose tolerance changes, and insulin secretion capacity following LD MAT2Ai was mild and similar in both groups (Figure 5, C–E), so the lower dose was chosen to perform the experiments and obtain data. Administration of LD MAT2Ai abolished differences between WT^HFD^ mice and SP^HFD^ groups with respect to kidney function and histology (Figure 5, F and G), supporting a model whereby decreased levels of *Mat2a* in PTCs of WT^HFD^ mice are important in progression of injury and play a causal role in tubular injury in metabolic syndrome, while preservation of *Mat2a* levels in PTCs of SP^HFD^ mice is protective.

### Methionine modulation controlled tubular phenotypic changes in DKD.

**Figure 6:**
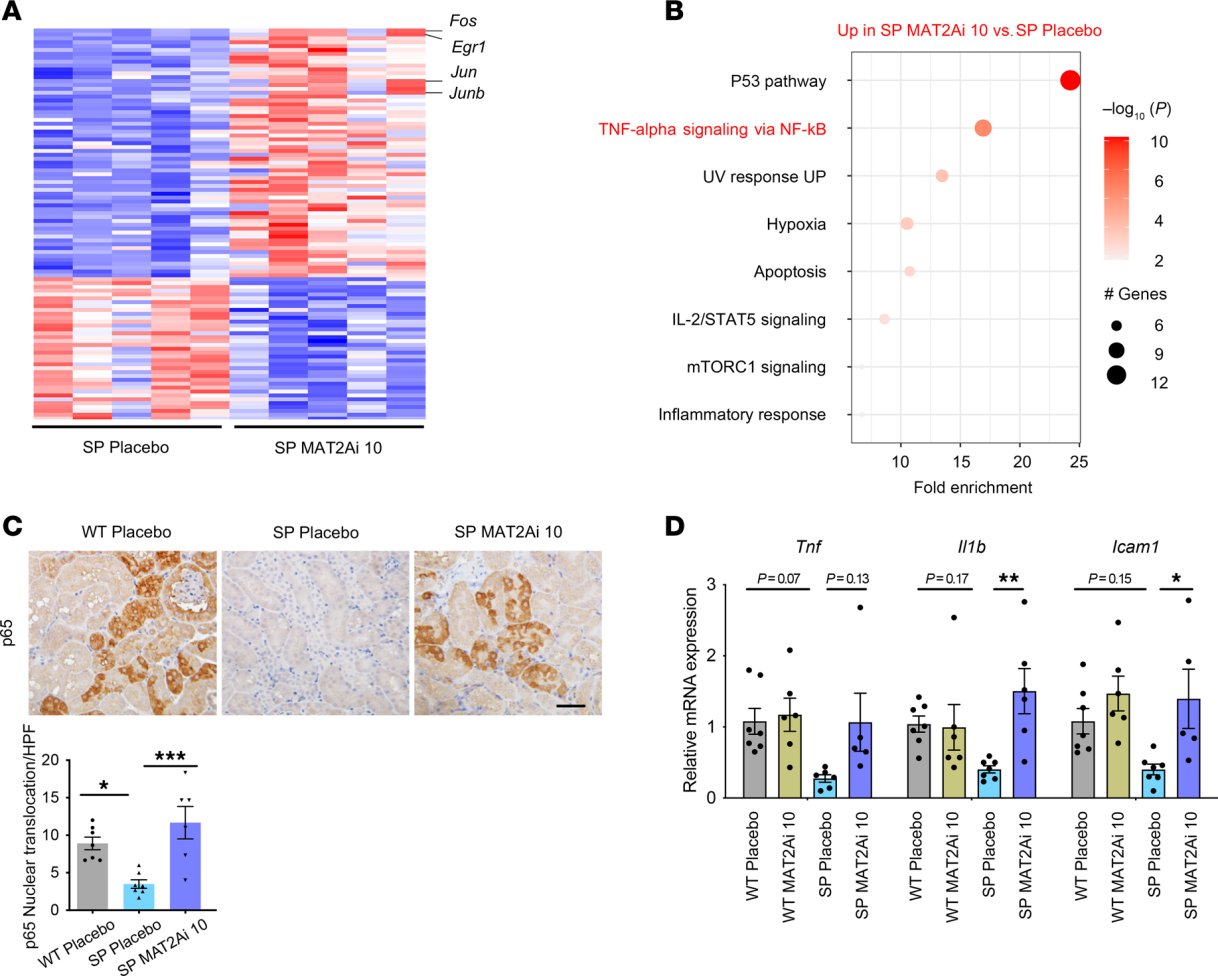
Inhibition of MAT2A modulates the tubular inflammatory phenotype in SP^HFD^ mice. (**A**) RNA-Seq heatmap for the top 50 differentially regulated genes. (**B**) Bubble plot of pathway enrichment; upregulated pathways in SP^HFD^-LD_MAT2Ai versus SP^HFD^ placebo (size of the dot indicates the percent positive cells, and color indicates relative expression). (**C**) Representative images of p65 staining across groups. Lower panel, quantification analysis. HPF, High-power field (original magnification ×40). (**D**) RT-qPCR analysis of proinflammatory cytokines and chemokines in tissue isolated from the renal cortex across groups. Sample numbers: WT-PL, *n* = 7; WT-MAT2Ai LD, *n* = 7; SP-PL, *n* = 8; SP-MAT2Ai LD, *n* = 8. Scale bar: 50 μm. One-way ANOVA; ***P < 0.001, **P < 0.01, and *P < 0.05 by Tukey’s test. Values are presented as mean ± SEM.

To further understand the role of SAM and methionine metabolism in the phenotypes observed in SP^HFD^ and WT mice fed HFD with or without MAT2Ai, we performed RNA-Seq analysis (Figure 6, A and B). MAT2Ai treatment led to a similar “injured” phenotype in SP^HFD^ mice as that observed in WT^HFD^ mice with increased NF-kB p65 nuclear translocation (Figure 6C) and increased expression of proinflammatory cytokines (Figure 6D). These results demonstrated that MAT2Ai treatment abolished the renoprotective effects of the LOF of SGLT2 and support a mechanism by which SGLT2 inhibition suppressed inflammatory response in a MAT2A-dependent manner.

**Figure 7:**
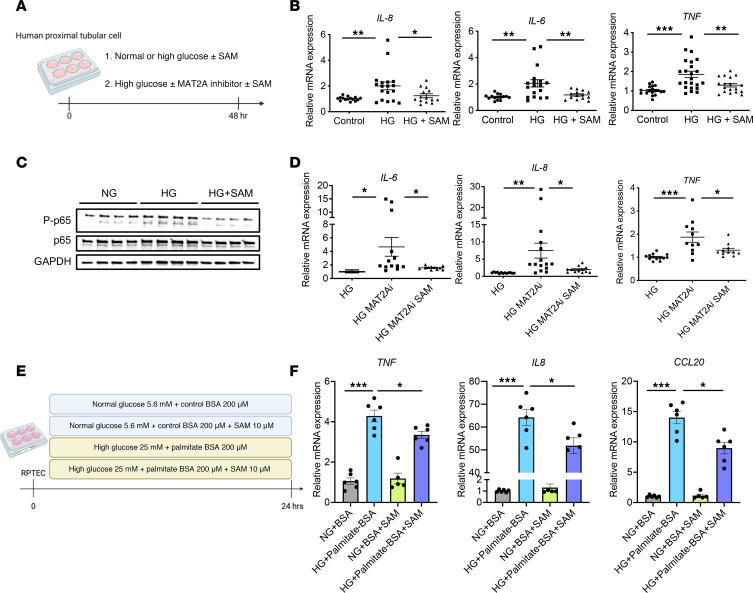
SAM supplementation inhibits HG-induced pathogenic phenotypes in human PTCs. (**A**) Schematic protocol of in vitro study using HK-2 cells. (**B** and **C**) SAM supplementation inhibited (**B**) transcript levels of proinflammatory cytokines (*n* = 18–24 cultures per group) and increased (**C**) phosphorylated p65 protein expression upon HG treatment. (**D**) MAT2Ai exacerbated proinflammatory cytokine expression, which are inhibited by SAM supplementation (*n* = 12–18 cultures per group). (**E**) Schematic protocol of in vitro study using RPTECs. (**F**) SAM supplementation inhibited transcript levels of proinflammatory cytokines in the presence of HG and palmitic acid (*n* = 6 cultures per group). One-way ANOVA; ***P < 0.001, **P < 0.01, and *P < 0.05 by Tukey’s test. Values are presented as mean ± SEM.

While inhibition of MAT2A is predicted to reduce SAM levels, we also wanted to test whether SAM supplementation is sufficient to rescue PTCs when exposed to high glucose (HG) and whether it can overcome the negative effect of the MAT2Ai. HG-treated, immortalized, renal PTCs (HK-2 cells) were treated with SAM for 48 h (Figure 7A). SAM supplementation prevented the upregulation of NF-κB–related genes and phosphorylation of NF-kB p65 induced by HG treatment (Figure 7, B and C). By contrast, MAT2Ai further exacerbated the injury phenotype in PTCs induced by HG treatment (Figure 7D). Strikingly, SAM treatment was able to prevent upregulation of inflammatory and injury markers in PTCs in vitro in the presence of both HG and MAT2Ai, supporting SAM as a key downstream product of MAT2A activity responsible for kidney protection (Figure 7D). To validate these findings in a more physiologically relevant context, we examined primary human renal proximal tubular epithelial cells (RPTECs) treated with HG and palmitic acid for 24 h to simulate HFD (Figure 7E). Similar to our observations in HK-2 cells, SAM treatment suppressed the induction of NF-κB downstream genes in RPTECs (Figure 7F). These data support a model whereby maintenance of the methionine pathway and, specifically, increased levels of the methionine metabolite SAM in PTCs play a role in renal protection seen with inhibition of SGLT2 function.

### Mice treated with an SGLT2 inhibitor phenocopied SGLT2 genetic LOF.

To determine whether the phenotype observed in SGLT2 LOF mice was similar to pharmacological inhibition of SGLT2, HFD-fed mice were treated with the SGLT2 inhibitor Canagliflozin (Cana). WT mice were prefed HFD for 4 weeks and subsequently administered placebo (0.5% hydroxypropyl methylcellulose [HPMC]) with or without Cana (20 mg/kg/day) via daily oral gavage for 8 weeks (Supplemental Figure 13A). Cana treatment successfully induced glucosuria (Supplemental Figure 13B). Although a transient reduction in BW was observed during the first 2 weeks of treatment, BW normalized over time due to compensatory hyperphagia (Supplemental Figure 13, C and D). Cana improved metabolic parameters by reducing postprandial hyperglycemia, glucose intolerance, and excessive insulin secretion induced by HFD (Supplemental Figure 13, E–G). Similar to the phenotype observed in SGLT2 LOF mice, Cana treatment increased kidney weight without affecting heart weight (Supplemental Figure 13, H and I). Moreover, Cana reduced TUNEL-positive cells, fibrosis, and proteinuria, although serum creatinine levels remained unchanged (Supplemental Figure 13, J–L).

scRNA-Seq analysis revealed that Cana suppressed the emergence of the PT-HFD subpopulation (Supplemental Figure 14A). Notably, gene expression patterns in PTCs from Cana-treated HFD mice closely resembled those from SP^HFD^ mice, with shared downregulation of NF-κB–related genes (Supplemental Figure 14, B and C). Consistent with this, reduced expression of *Fos* and *Il1b* was observed in PTCs from Cana-treated mice (Supplemental Figure 14D), along with diminished nuclear translocation of NF-κB p65 (Supplemental Figure 14E). Collectively, these findings demonstrate that pharmacological inhibition of SGLT2 recapitulates the anti-inflammatory effects of genetic SGLT2 loss through repression of the NF-κB pathway.

### Enhanced repressive trimethylation of H3K27 in inflammatory pathway genes following HFD in mice with SGLT2 LOF.

**Figure 8:**
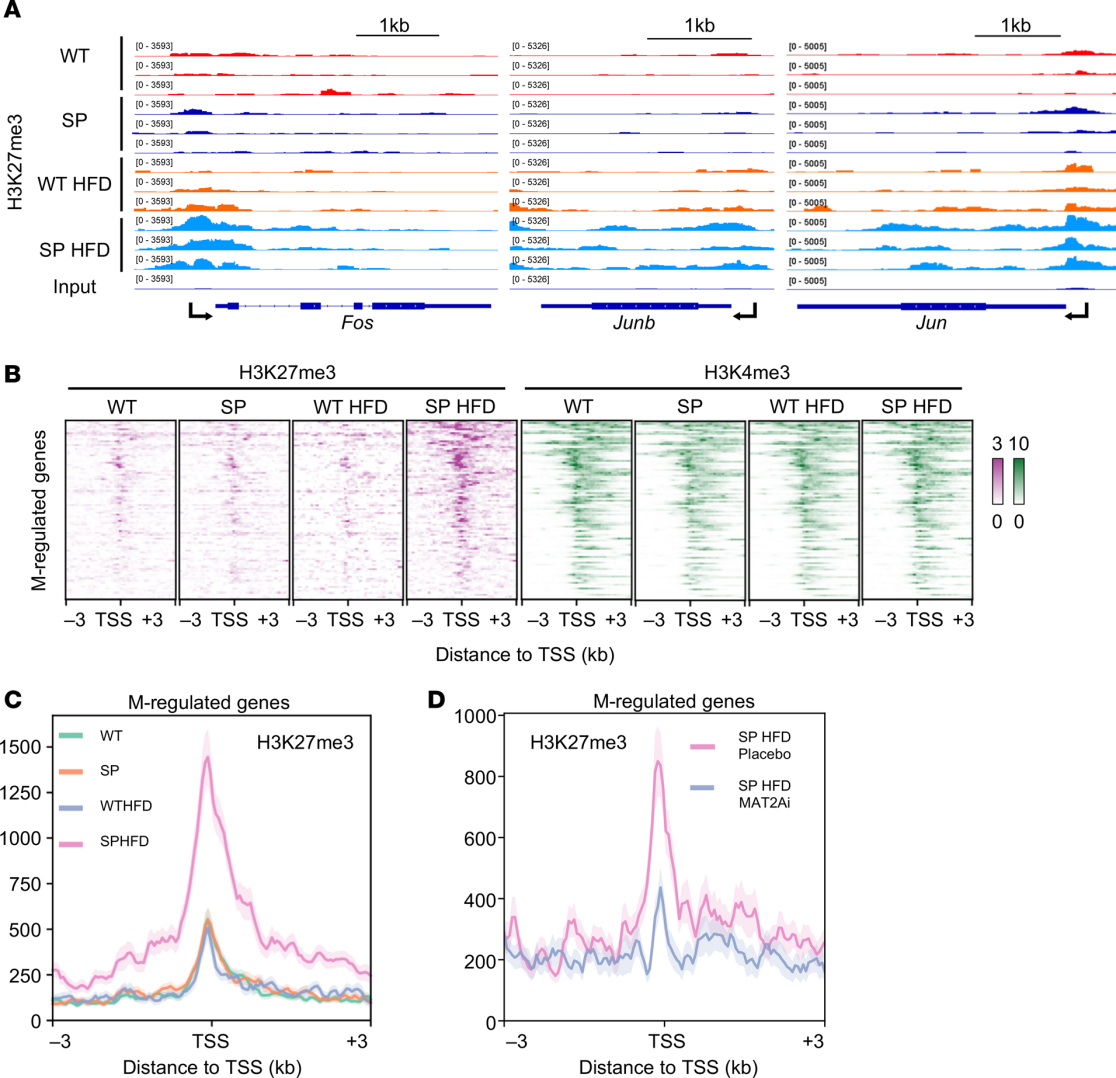
SP^HFD^ mice exhibit increased repressive histone modification at M-regulated gene promoters. (**A**) Tracks for *Fos*, *Junb*, and *Jun* of H3K27me3 CUT&RUN in the indicated mouse renal cortex. Data from 3 mice for each condition are shown. The *y* axis indicates reference-normalized reads per million (RRPM). (**B**) Heatmap showing CUT&RUN signal for H3K27me3 and H3K4me3 at M-regulated genes. Signal is centered on the TSS. The scale of signal is shown as RRPM × 10^3^. *n* = 90. (**C** and **D**) Meta-profiles of CUT&RUN H3K27me3 signal at the M-regulated genes. The *y* axis indicates the mean H3K27me3 signal (RRPM). *n* = 90 (**C**) and 2,924 (**D**).

SAM regulates chromatin dynamics, serving as the cosubstrate for methyl donor transfers to histones (21). This led us to hypothesize that the elevated levels of SAM in kidneys from mice with LOF of the SGLT2 cotransporter result in histone methylation changes that underlie the molecular signatures associated with kidney protection in metabolic syndrome. To test this hypothesis, we performed cleavage under targets and release using a nuclease enzyme (CUT&RUN) (34) in renal cortex from WT^HFD^ and SP^HFD^ mice and profiled genome-wide trimethylation in H3K27 and H3K4, markers of inactive and active regions of the genome, respectively. Intriguingly, H3K27me3 was enriched at the transcription start sites (TSSs) in promoters of NF-κB cooperative factors (*Fos*, *Jun*, *Junb*, and *Ecsit*), NF-κB components (*Nfkb1*, *Nfkb2*, *Rela*, and *Relb*), and downstream targets such as *Icam1* in SP^HFD^ (Figure 8A and Supplemental Figure 15), providing a mechanism for repression of proinflammatory genes. If our model is correct, we also predicted that genes increased in SP^HFD^ mice treated with MAT2Ai to reduce SAM production should represent the subset of genes that are regulated by changes in methionine metabolism and SAM within the kidney. We refer to this subset of genes as methionine pathway–regulated (M-regulated) genes (Figure 6A). To test this hypothesis, we next compared H3K27me3 levels at TSSs of genes in this dataset (M-regulated genes) in SP^HFD^ mice.

In support of the model, CUT&RUN data showed a deposition of the suppressive mark H3K27me3 at the TSSs of many of these M-regulated genes in renal cortex isolated from SP^HFD^ mice but not in WT^HFD^ mice (Figure 8, B and C, and Supplemental Figure 16A). Importantly, H3K27me3 was mostly unchanged at the regions with broad peaks, where this mark is also present in WT mice (Supplemental Figure 16, B and C). This result suggests that H3K27me3 in SP^HFD^ was modulated in a gene-specific manner. However, the active histone mark H3K4me3 remained stable across all groups (Figure 8B and Supplemental Figure 16A), suggesting that the SP genotype did not impact changes at this activation mark. By contrast, deposition of H3K27me3 at TSSs of M-regulated genes in SP^HFD^ mice was not found in SP^HFD^ mice treated with MAT2Ai (Figure 8D and Supplemental Figure 16C). To elucidate the mechanisms underlying the given selectivity of H3K27me3 deposition, we examined the expression of components of Polycomb repressive complex2 (PRC2), a histone methyltransferase responsible for mono-, di-, and trimethylated H3K27 (35). Among them, we have identified increased levels of Aebp2, a cofactor of PRC2, at transcript and protein levels in the PTCs or renal cortex (SP^HFD^ > WT^HFD^) (Supplemental Figure 17, A and B).

**Figure 9:**
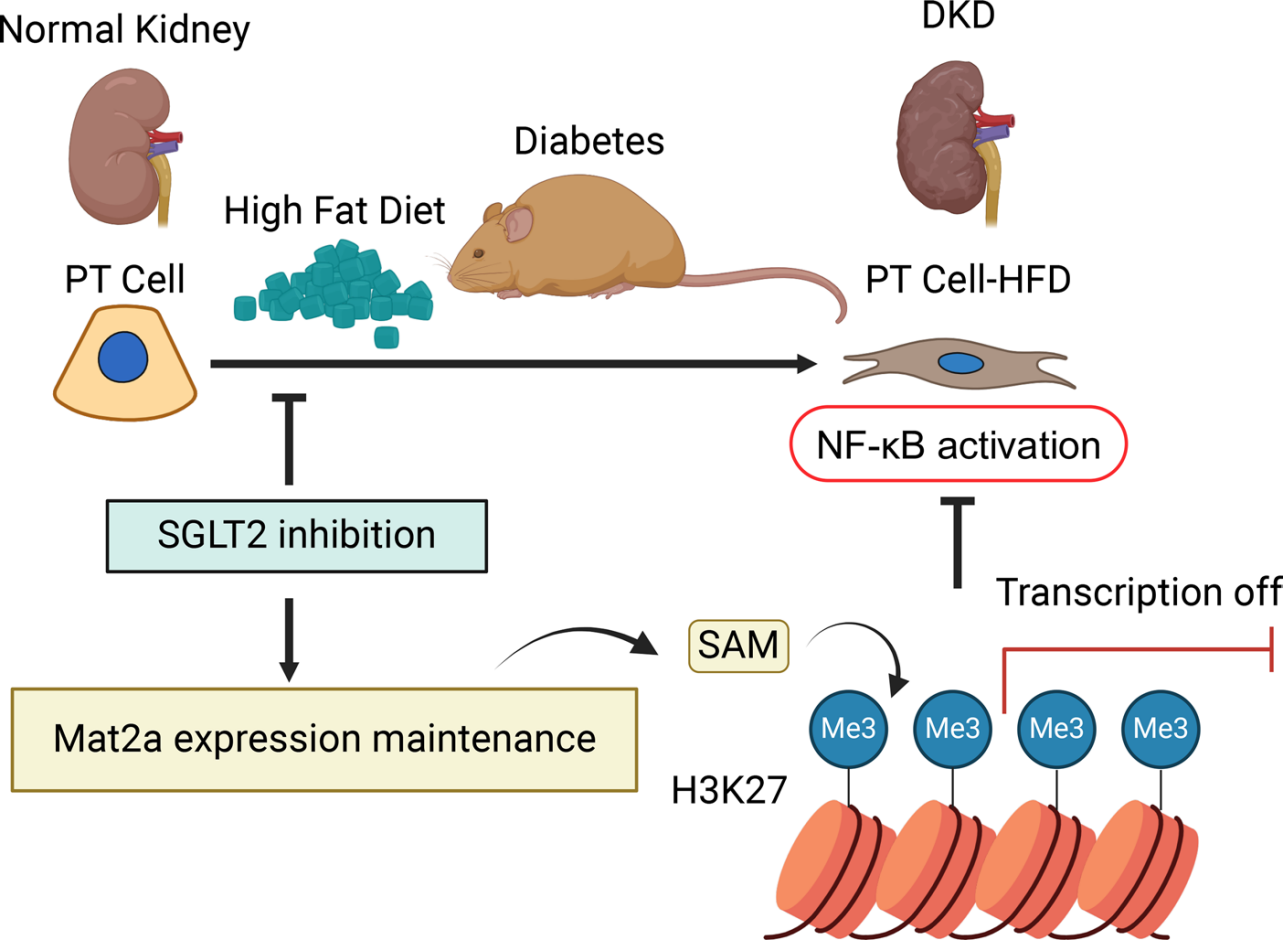
Working model for SGLT2 kidney protection under HFD metabolic stress. Straight arrows indicate the direction of progression. T-shaped arrows indicate inhibition.

Altogether, these results support a model whereby reduced inflammation observed in SP^HFD^ mice was regulated through epigenetic modulation of H3K27me3 that was associated with elevated levels of the SAM metabolite due to higher levels of *Mat2a*. Thus, we posit that the region-specific enhancement of repressive histone methylation observed in mice with SGLT2 LOF regulated pathogenic gene expression in the setting of metabolic stress (Figure 9).

## Discussion

In recent years, several excellent studies have been published providing mechanisms of kidney protection beyond glucosuria and glucose control afforded by the SGLT2 inhibitors (14, 15, 18). Given the pleiotropic effects of SGLT2 inhibitors, it remains difficult to fully exclude the possibility that their kidney protective effects are mediated, at least in part, through improved insulin resistance as a consequence of better glucose control. However, to date, no studies have focused on metabolites that are altered following SGLT2 inhibition or genetic LOF and whether they are functionally important. Our study sought to bridge this gap and focused on the role of metabolites and whether they provide benefit at the earliest stages of metabolic disease. Metabolites have emerged as key drivers of cellular function and cellular processes (20). Here, we speculated that blocking glucose uptake in proximal tubular epithelial cells results in altered renal metabolites, which trigger cellular events that protect the kidney. Using metabolomics, we identified increased levels of SAM in kidneys of mice that lack a functional PTC-associated SGLT2 cotransporter. Given the principal role of SAM as a methyl donor, we further hypothesized that elevation of this metabolite would drive epigenetic changes that underpin transcriptional changes associated with kidney injury in early stages of a diabetogenic setting (e.g., HFD).

To address these hypotheses, we used the SP mouse model (22). Benefits of this model include: (a) the ability to study on-target effects of SGLT2 inhibition in isolation and (b) the ability to block 100% activity without concern for pharmacokinetic availability of a pharmacologic treatment. The scRNA-Seq data from kidney confirmed that *Slc5a2* is only expressed in S1 and S2 segments of the proximal tubule in the mouse kidney. We also confirmed that kidneys of the genetic LOF SP model were protected from HFD similar to studies performed with pharmacologic SGLT2 inhibitors (18) and that gene pathways altered in PTCs and kidneys of WT mice fed HFD were similar to those observed in patients and mice with both DKD and CKD (10, 12, 14, 36, 37). Comparison of our data with published human datasets in kidneys isolated under diabetic conditions (27) showed early in the course of disease, a subcluster of PTCs emerged in WT^HFD^ mice that assumed an injury phenotype, similar to those observed in humans and that are characterized by genes associated with increased NF-κB activation.

Most prominent and consistent in our dataset and across published datasets from patients, expression of the gene encoding the SAM synthetase, *Mat2a*, was decreased in injured PTCs that emerge during the course of kidney disease triggered by metabolic stress (e.g., HFD) compared with other PTCs. MAT2A, is a key enzyme in the methionine metabolic pathway that is required for the generation of SAM, a metabolite that functions as a methyl donor (21). Because decreased expression of *Mat2a* or blockade of MAT2A with an inhibitor is predicted to lead to decreased levels of the SAM metabolite in injured PTCs, and elevated SAM levels are associated with renal protection in mice and proximal tubule cells in vitro, we focused on determining the mechanism(s) whereby SAM provides benefits. As SAM functions as a methyl donor, we speculated that the increased levels of SAM in SP^HFD^ mice compared with WT^HFD^ mice might result in epigenetic changes leading to “protective” gene signatures. To determine if this might be the case in the kidney, we performed CUT&RUN from kidney tissue samples of WT and SP mice under ND and HFD conditions. While H3K27 methylation was increased in both HFD-fed groups, it was more greatly increased in SP mice. To determine if there was any specificity or direct link to transcriptional changes we observed, we profiled genome-wide trimethylation in H3K27 and H3K4, markers of inactive and active regions of the genome, respectively. We found an association between enhanced H3K27 trimethylation at TSS of genes related to the NF-κB pathway, providing a mechanism whereby SGLT2 inhibitors lead to decreased expression of these proinflammatory, pro-injurious pathways. Furthermore, the lack of change in H3K27 broad peaks that were present across all groups supports a specific/functional/causal role for methylation in regulating disease-associated pathways. Finally, treatment with MAT2Ai showed that protection of kidneys was lost in SP^HFD^ mice, and repeated CUT&RUN on tissue samples harvested from these mice showed loss of H3K27 trimethylation, especially at TSSs of genes that include NF-κB pathway target genes. We posit this effect is likely driven by a reduction in SAM levels, which we confirmed were reduced in kidney tissue harvested from mice treated with a MAT2Ai. However, it remains possible that additional effects of MAT2Ai impact differences between groups, which were not fully explored here. Our metabolomic analysis also revealed enhanced taurine and cysteine metabolism in the SP^HFD^ group. Given their well-established antioxidant properties, both taurine, cysteine, and their respective metabolites may contribute to renal protection under metabolic stress (38, 39).

There are limitations of our study. The mechanism by which SGLT2 inhibition alters methionine metabolism remains unclear. Given that SAM and glucose metabolism are interconnected through the 1-carbon metabolic network (40), investigating this link is of particular interest. In vivo glucose tracing experiments will be helpful to determine how glucose availability or flux influences methionine cycle activity in SP mice. Additionally, previous studies have implicated both mTORC1 signaling and the long noncoding RNA *Malat1* in the regulation of *MAT2A* expression (41, 42). In our scRNA-Seq dataset, expression of *mTOR* and *Malat1* was elevated in PTCs of SP mice (data not shown), suggesting a plausible mechanistic axis that warrants further investigation. Future studies should examine the human relevance of our findings. To elucidate the roles of PTC-specific SAM modulation in vivo and assess potential off-target effects of MAT2Ai, the conditional MAT2A-knockout mice could be generated and studied, although complete loss of MAT2A activity is predicted to have a severe phenotype and perhaps uninterpretable results. The number of PT-HFD cells and their heterogeneous cell state make their identification in tissue sections challenging. Also, determining the localization of SAM within cells/tissues in vivo would be helpful, but it is currently beyond available tools (spatial metabolomics was attempted but unsuccessful). While the current data provide strong support for an association between SAM elevation, H3K27me3, and repression of inflammatory genes, it is not possible to exclude other mechanisms for gene regulation by the SAM metabolite. Future studies using H3K27me3 inhibitors and/or mutants of the PRC2 enzyme are needed to confirm a causal relationship.

Taken together, we have identified modulation of methionine metabolism in kidneys of HFD-fed mice and identified protective methylation in mice lacking the SGLT2 cotransporter. These data support a model and mechanism by which an altered metabolite directly drives cellular responses to prevent injury.

## Methods

### Sex as a biological variable.

For studies involving animal models, male mice were used because male animals are known to exhibit less variability in phenotype. However, the single-nucleus RNA sequencing analyses included datasets that included both male and female human samples as well as female *ob*/*ob* mice.

### Mouse models.

SGLT2 mutant (MT) and WT C3H/HeJ (C3H) male mice (10 weeks old) were fed a HFD (60% calories from fat) (TD.06414; Envigo) or ND for 8 or 18 weeks. For MAT2Ai treatment, WT and MT mice were fed a HFD for 8 weeks and then treated with placebo (DMSO) or 2 dosages of (*E*)-4-(2-chloro-6-fluorostyryl)-*N*-methylaniline (A1328405; Ambeed) (MAT2Ai, placebo, 10 or 50 mg/kg BW) (41, 43) through the i.p. route 3 times per week for 8 weeks. To evaluate the effects of Cana under metabolic stress, 10-week-old male C3H mice were fed HFD for 4 weeks. After this pretreatment period, mice were assigned to receive either daily oral administration of Cana (20 mg/kg/day) or placebo control (0.5% HPMC) by gavage for an additional 8 weeks. Cana was suspended in 0.5% HPMC before administration. All treatments were administered once daily in the morning. BW and food intake were monitored weekly throughout the treatment period.

### Genotyping and detection of urinary glucose excretion.

Genomic DNA was isolated from mouse tails, and PCR was used to identify SP mutants with the following primers to produce a 470 bp PCR product: sense 5′-TGTGAGGCTGTCCCAAGAATGT-3′ and antisense 5′-TCAGAGTCCCAGCATTTGGTCT-3′. The PCR product was then digested with restriction enzyme TaqI (T↓CGA) for 90 min at 65°C (WT: 2 bands at 225 and 245 bp; heterozygous mutant: 3 bands at 225, 245, and 470 bp; homozygous mutant: 1 band at 470 bp). The urinary glucose excretion was determined using urine strips (11895362160; Roche Diagnostics) (Supplemental Figure 1A).

### Cell culture.

HK-2 human PTCs were procured from the American Type Culture Collection (CRL-2190; ATCC) and grown in Keratinocyte serum-free medium (17005-042; Gibco) at 37°C and 5% CO_2_. Cells were stimulated with normal glucose (5.6 mM) or high glucose (50 mM) DMEM supplemented with 1% FBS treated with/without SAM (A7007; Sigma-Aldrich) (1 μM) and/or MAT2Ai (A1328405; Ambeed) (1 μM) supplementation for 48 h. RPTECs (CC-2553; Lonza) were cultured in renal epithelial cell growth medium (CC-3190; Lonza). RPTECs were stimulated with normal glucose (5.6 mM) or HG (50 mM) DMEM, supplemented with 1% FBS, and treated with 200 μM BSA Control (295565; Cayman Chemical) or BSA-Palmitate (29558-5; Cayman Chemical) in the presence or absence of SAM (10 μM; A7007; Sigma-Aldrich) for 24 h.

### Preparation of single-cell suspension.

Kidneys from male SGLT2 WT/MT mice fed with ND or HFD for 8 weeks were harvested. Kidneys were minced with a sterile razor blade into small pieces (<1 mm in diameter) after removing capsule. Approximately 65 mg tissue was digested in 1.5 mL digestion buffer including Liberase TM (0.3 mg/mL; 291963; Roche), hyaluronidase (10 μg/mL; #H4272, Sigma-Aldrich), and DNase I (20 μg/mL; 11284932001; Roche) at 37°C for 40 min on a shaker (300 rpm, The Eppendorf Thermomixer 5350). After centrifugation, the cell pellet was resuspended in 2 mL red blood cell lysis buffer (00-4333-57; Thermo Fisher Scientific) and incubated for 2 min at room temperature. Cells were then further digested with 5 mL 0.25% trypsin EDTA (Gibco) at 37°C for 30 min on a shaker (100 rpm, Thermo Scientific MaxQ 6000 incubated/refrigerated stackable shakers) after washing with 0.04% BSA in PBS 3 times. Trypsin was inactivated using 2 mL 10% FBS in PBS after digestion. Cells were then washed with 10% FBS twice and 0.04% BSA twice followed by resuspension in ice-cold PBS supplemented with 0.04% BSA. After filtration through a 40 μm strainer, cell number and viability were analyzed using the Nexcelom Cellometer Auto2000 with the acridine orange (AO) and propidium iodide (PI) fluorescent staining method. Ten thousand cells for each group were loaded into the Chromium Controller (10X Genomics) on a Chromium Next GEM Chip G (10X Genomics) and processed to generate single-cell gel beads in the emulsion (GEM) according to the manufacturer’s protocol. The cDNA and library were generated using the Chromium Next GEM Single Cell 3′ Reagent Kits v3.1 (10X Genomics) and Single Index Kit T Set A (10X Genomics) according to the manufacturer’s manual. Quality control (QC) for constructed library was performed using a Bioanalyzer High Sensitivity DNA Kit (Agilent Technologies) and a Qubit DNA HS assay kit for qualitative and quantitative analysis, respectively. The multiplexed libraries were pooled and sequenced on an Illumina HiSeq 4000 sequencer with 2 × 50 paired-end reads using the following read length: 28 bp Read1 for cell barcode and UMI (unique molecular identifier), 8 bp I7 index for sample index, and 91 bp Read2 for transcript.

### scRNA-Seq.

Cell Ranger (v1.0.0) was used for demultiplexing and counting. The R (R Core Team, 2023) (44) package Seurat (v4.0.0) (45) was used for data preprocessing and visualization. Initially, droplets with less than 200 or more than 5,000 feature counts were removed. Genes expressed in fewer than 3 cells were discarded. As renal epithelial cell types have high metabolic rates and are very rich in mitochondria, cells were further filtered on the basis of percentage of reads mapping to mitochondrial genes less than 50%. Doublets were identified using a 2-layer approach, as described previously: first, scDblFinder (v1.4.0) (46) was used to predict potential doublets using default settings in an automated and unbiased fashion. Doublets were additionally identified manually when expressing combinations of marker genes from different cell types. After doublet removal, single cells were normalized with NormalizeData and then integrated using an anchor-based canonical correlation analysis (CCA) pipeline. The first 50 principal components were used for clustering, and the resolution was set at 0.5. Uniform Manifold Approximation and Projection was used for cluster visualization. Marker genes for each cluster were identified by the FindAllMarkers function. We detected 30 unique clusters. Cell identity to each cluster was assigned based on previously published canonical marker genes (13, 47–50). Differential gene expression analyses of the PT-HFD were performed using the FindMarkers function based on Wilcoxon’s rank sum test. A threshold of 0.25 for log fold change and 0.05 for the adjusted *P* value were applied for downstream analysis.

### Pathway enrichment analysis.

Pathway enrichment analysis were performed using Enrichr (51) to identify potential pathways and molecular functions affected in PT-HFDs, and the retrieved combined score (log(*P* value) * *z* score) was displayed.

### KPMP dataset analysis.

KPMP dataset analysis was based upon data generated by the Kidney Precision Medicine Project (https://www.kpmp.org; accessed December 27, 2024) funded by the National Institute of Diabetes and Digestive and Kidney Diseases (grants U01DK133081, U01DK133091, U01DK133092, U01DK133093, U01DK133095, U01DK133097, U01DK114866, U01DK114908, U01DK133090, U01DK133113, U01DK133766, U01DK133768, U01DK114907, U01DK114920, U01DK114923, U01DK114933, U24DK114886, UH3DK114926, UH3DK114861, UH3DK114915, and UH3DK114937).

### Metabolomics in serum and renal cortex from SGLT2 WT or MT mice.

The metabolomic analysis was conducted by Metabolon Inc.

Samples were prepared using the automated MicroLab STAR system from Hamilton Company. Several recovery standards were added prior to the first step in the extraction process for QC purposes. To remove protein, small molecules bound to protein or trapped in the precipitated protein matrix were dissociated, and to recover chemically diverse metabolites, proteins were precipitated with methanol under vigorous shaking for 2 min (Glen Mills GenoGrinder 2000) followed by centrifugation. The resulting extract was divided into 5 fractions: 2 for analysis by reversed phase ultra-performance liquid chromatography tandem mass spectrometry with positive ion mode electrospray ionization (ESI), 1 for analysis by RP/UPLC-MS/MS with negative ion mode ESI, 1 for analysis by hydrophilic interaction liquid chromatography/UPLC-MS/MS with negative ion mode ESI, and 1 sample was reserved for backup. Samples were placed briefly on a TurboVap evaporator (Zymark) to remove the organic solvent. The sample extracts were stored overnight under nitrogen before preparation for analysis. Several types of controls were analyzed in concert with the experimental samples: a pooled matrix sample generated by taking a small volume of each experimental sample (or a pool of well-characterized human plasma) served as a technical replicate throughout the dataset; extracted water samples served as process blanks; and a cocktail of QC standards that were carefully chosen not to interfere with the measurement of endogenous compounds were spiked into every analyzed sample, allowing instrument performance monitoring and aiding chromatographic alignment. Instrument variability was determined by calculating the median relative standard deviation (RSD) for the standards that were added to each sample prior to injection into the mass spectrometers. Overall process variability was determined by calculating the median RSD for all endogenous metabolites (i.e., non-instrument standards) present in 100% of the pooled matrix samples. Experimental samples were randomized across the platform run with QC samples spaced evenly among the injections. The informatics system consisted of 4 major components, the Laboratory Information Management System, the data extraction and peak identification software, data processing tools for QC and compound identification, and a collection of information interpretation and visualization tools for use by data analysts. The hardware and software foundations for these informatics components were the LAN backbone and a database server running Oracle 10.2.0.1 Enterprise Edition. Raw data were extracted, peak identified, and QC processed using Metabolon’s hardware and software. Compounds were identified by comparison with library entries of purified standards or recurrent unknown entities. The authenticated standards contained the retention time/index (RI), mass-to-charge ratio (*m*/*z*), and chromatographic data (including MS/MS spectral data) on all molecules present in the library. Furthermore, biochemical identifications were based on 3 criteria: RI within a narrow window of the proposed identification, accurate mass match to the library ±10 ppm, and the MS/MS forward and reverse scores between the experimental data and authentic standards. The MS/MS scores were based on a comparison of the ions present in the experimental spectrum to the ions present in the library spectrum. While there may be similarities between these molecules based on 1 of these factors, the use of all 3 data points allows the biochemicals to be distinguished and differentiated. Peaks were quantified using AUC. For studies spanning multiple days, a data normalization step was performed to correct variation resulting from instrument interday tuning differences. Essentially, each compound was corrected in run-day blocks by registering the medians to equal 1.00 and normalizing each data point proportionately. Significantly altered metabolites (*P* < 0.05) were used to perform metabolite set enrichment analysis by MetaboAnalyst software (version 5.0) (52).

### Glucose tolerance and insulin tolerance test.

Glucose tolerance tests were performed in mice fasted for 16 h. Blood glucose and insulin were measured at the indicated times following i.p. glucose injection of 2 g/kg BW. Insulin tolerance tests were performed after a 2 h fast, followed by i.p. injection of 0.75 U insulin/kg BW (Humulin R; Eli Lilly). Plasma was collected by centrifugation at 850*g* for 20 min, and plasma insulin levels were determined by ELISA (90080; Crystal Chem Inc.). Blood glucose level was measured by a Contour blood glucose meter (Bayer) (53).

### uACR measurement.

uACRs were determined by ELISA (Albuwell M kit, 1011; Ethos Bioscience) and the microtiter-format colorimetric Jaffe reaction assay using alkaline picrate (54).

### Serum creatinine measurement.

Mouse serum was collected by centrifugation at 850*g* for 20 min. Serum creatinine levels were measured by isotope dilution liquid chromatography–MS/MS (LC-MS/MS) (55) at University of Alabama at Birmingham and University of California San Diego O’Brien Center Core C using an Agilent Infinity 1260 LC and an Infinity 1290 autosampler with a 6460 Triple Quad mass spectrometer as well as a TSK-Gel Amide-80 column (Tosoh Bioscience). Separation was achieved with an isocratic flow of 10 mM ammonium acetate in 65% acetonitrile. For LC-MS/MS determinations, 5 to 50 μL of sample was deproteinated and diluted with heavy isotope-labeled internal standard (ISTD) in a single step by adding ISTD in 80% acetonitrile. Two microliters of diluted sample was subjected to isocratic, HILIC HPLC with 10 mM ammonium acetate in 65% acetonitrile at 0.15 mL/min. Creatinine and d3-creatinine (ISTD) were detected by ESI MS/MS multiple reaction monitoring transitions 114 > 44 and 117 > 47, respectively. Quantitation was achieved by comparing results with a synthetic standard calibration curve (0, 0.2, 1, 5, and 100 μg/mL for serum).

### RT-qPCR.

RNA was isolated from various organs using the standard Trizol (15596018; Life Technologies) extraction protocol. cDNA was reverse transcribed using the iSCRIPT synthesis kit (1708891; Bio-Rad). The cDNA was used as the template for RT-qPCR, which was performed with Fast SYBR Green Master Mix (Applied Biosystems) on a QuantStudio 3 Real-Time PCR System (Thermo Fisher Scientific) (54). GAPDH or hypoxanthine phosphoribosyltransferase 1 was used as the internal control for the reactions. Relative gene expression levels were calculated with the comparative Ct (2DDCt) method. Primer sequences are listed in Supplemental Table 4.

### Immunoblotting.

Cells and mouse kidney tissues were lysed with radioimmunoprecipitation buffer supplemented with Halt protease and phosphatase inhibitor cocktails (78442; Thermo Fisher Scientific) on ice for 30 min. Protein concentration in the lysates was measured with a bicinchoninic acid assay (Thermo Fisher Scientific). Equal amounts of protein in Laemmli sample buffer composed of 1% SDS, 62.5 mM Tris-HCl (pH 6.8), 10% glycerol, 5% β-mercaptoethanol, and 0.05% bromophenol blue were boiled for 10 min, separated by SDS-PAGE, and transferred to a PVDF membrane (GE Healthcare), which was blocked with 5% BSA in Tris-buffered saline and 0.1% Tween 20 (TBST) for 1 h at room temperature, followed by overnight incubation at 4°C with primary antibodies. After 3 washes with TBST, the membrane was incubated with appropriate HRP-conjugated secondary antibodies for 1 h at room temperature. The antigen was detected with a SuperSignal West Pico PLUS Chemiluminescent Substrate (34580; Thermo Fisher Scientific) according to the manufacturer’s instructions. Information on antibodies is provided in Supplemental Table 5.

### Immunofluorescence.

Frozen kidney sections (5 μm) were dried at room temperature for 1 h, and the frozen section was fixed with precooled acetone (–20°C) for 10 min. After blocking with 10% normal horse serum for 60 min, the tissue was incubated overnight at 4°C with primary antibody (fibronectin; 1/200). Alexa-labeled secondary antibody (1/300) was used for signal detection. For immunofluorescent imaging, confocal microscopy was performed using a Nikon A1 confocal microscope from the Center of Advanced Microscopy at Northwestern University. Different sample images of the same antigen were acquired under constant acquisition settings. For image analysis, Fiji software was used (56). Information on antibodies is provided in Supplemental Table 5.

### Histological analysis.

Tunnel staining was performed by the Northwestern University Mouse Histology and Phenotyping Laboratory. Sirius Red staining was performed to evaluate the degree of fibrosis. For immunohistochemical detection of NF-kB p65, kidney tissue was fixed with 10% buffered formalin phosphate (Fisher Scientific), and sections cut at a thickness of 5 μm were incubated with the appropriate primary antibody (57). The signal was detected by incubation with ImmPACT diaminobenzidine peroxidase substrate (SK-4105; Vector Laboratories). Three randomly selected fields were scored, and the average score was calculated. Glomerulosclerosis scoring was performed on PAS-stained sections using light microscopy at ×60 magnification. For each mouse, the glomerular injury index was calculated as the mean score of 20 glomeruli. Glomerulosclerosis was graded on a scale from 0 to 4 (58, 59). For image analysis, Fiji software was used (56). Information on antibodies is provided in Supplemental Table 5.

### Bulk RNA-Seq analysis.

Total RNA was extracted from kidney cortex using an RNeasy mini kit (74104; Qiagen), followed by genomic DNA digestion with DNase (79254; Qiagen). Poly(A) mRNA isolation and library preparation were performed using the Poly(A) mRNA Magnetic Isolation Module (E7490; New England Biolabs) and the NEBNext Ultra II Directional RNA Library Prep Kit (E7760; New England Biolabs). The libraries were sequenced on a NovaSeq 6000 (Illumina). Raw sequencing data underwent QC assessment using FastQC. Adapters and low-quality bases (Phred score < 33) were removed. Cleaned reads were aligned to mouse genome mm10 using STAR (v2.5.2) (60). Gene expression levels were quantified from the aligned reads using htseq counts with the reference annotation from mm10.Ens.78. Identification of differentially expressed genes was performed using the edgeR (v4.0.1) package (61) in R with a significance threshold of adjusted *P* value < 0.05. Principal component analysis (PCA) plot with raw counts was generated by the ggplot2 (v3.4.4) R package. The PCA plot with raw counts and the bubble chart showing pathway enrichment analysis were generated by the ggplot2 (v3.4.4) R package.

### CUT&RUN library preparation and peak calling.

CUT&RUN assay libraries for mouse kidney cortex were generated using the CUT&RUN Assay Kit (86652; Cell Signaling) according to the manufacturer’s instructions. Nuclei were isolated from frozen mouse kidney using nuclei isolation buffer (NUC-101; Sigma-Aldrich). Obtained nuclei were then mixed and incubated with concanavalin A–conjugated paramagnetic beads. Antibodies or rabbit IgG negative control were added to each sample. The remaining steps were performed according to the manufacturer’s instructions, followed by DNA purification using ChIP DNA Clean & Concentrator (D5205; Zymo Research). This purified DNA was used to prepare libraries using the KAPA Hyper prep kit (Roche). Libraries were sequenced on the NovaSeq 6000. Low-quality bases and adapters were removed from the 3**′** end using Trimmomatic 0.39 (62). Paired-end reads were aligned to a concatenated genome consisting of mouse mm10 and spike-in yeast sacCer3 assemblies using bowtie 2.4.5 (63) with --very-sensitive-local and --dovetail options. The aligned reads with mapping quality (MAPQ) < 20 were removed. To generate the genome browser tracks in bigwig format, the fragment counts were normalized to total reads aligned to the spike-in genome using deepTools 3.5.1 bamCoverage (64). Tracks from bigwig files were visualized using IGV2.12.2 (Broad Institute). Peak calling for broad H3K27me3 regions was performed using epic2 (65). Heatmaps and meta-profiles were generated using deepTools 3.5.1. SAM-regulated genes were defined as upregulated in SPMAT2Ai compared with SP-PL with a significance threshold of adjusted *P* value < 0.1. Information on antibodies is provided in Supplemental Table 5.

### In situ hybridization (RNAScope).

In situ hybridization was performed on the paraffin sections using RNAscope and following ACD protocol (https://acdbio.com/rnascope-25-hd-duplex-assay; probe: RNAscope Probe- Mm-Fos-C3; 316921-C3).

### Statistics.

Data are expressed as mean ± SEM and are representative of at least 3 independent experiments. Following the Grubbs’ outlier test, data were analyzed by 1- or 2-way ANOVA or by the 2-tailed Student’s *t* test with a significant difference defined as *P* < 0.05. The 1- or 2-way ANOVA was corrected for multiple comparisons with Tukey’s test. Statistical analyses were performed using Prism 6 and 10 software (GraphPad Inc.), and Grubbs’ outlier test was performed using the software on the GraphPad Inc. website.

### Study approval.

Mouse handling, husbandry, and all surgical procedures were performed under the approval of the IACUC of Northwestern University (approval number IS00002939).

### Data and code availability.

Data in this study are publicly available in the National Center for Biotechnology Information’s Gene Expression Omnibus under accession number GSE268597. This paper does not report original code. Any additional information is available upon reasonable request from the corresponding author. Values for all data points in graphs are reported in the Supporting Data Values file.

## Author contributions

HM, NSC, and SEQ designed the study. HM performed the majority of the experiments. YA, HK, ZLS, RPC and BM provided technical advice. ZLS, RPC, MEF, and DSK provided technical support. PG performed the quantitative LC-MS/MS analysis. HM and YZ analyzed omics data. HM, YA, BCH, GA, and YZ performed CUT&RUN and analysis of the data. HM wrote the manuscript, and YZ, YA, PK, JB, AS, NSC, and SEQ provided conceptual advice. HM, YZ, YA, PK, JB, AS, NSC, and SEQ edited the manuscript. All authors approved the manuscript.

## Supplementary Material

Supplemental data

Unedited blot and gel images

Supplemental table 1

Supplemental table 2

Supplemental table 3

Supporting data values

## Version 1 10/01/2025

Electronic publication
