# Supplementary material for: SGLT2 inhibition protects kidney function by SAM-dependent epigenetic repression of inflammatory genes under metabolic stress
Source: J Clin Invest. 2025 Oct 1;135(19):e188933. doi: 10.1172/JCI188933 (PMC12483609; doi:10.1172/JCI188933)
Supplement: Supplemental data [file jci-135-188933-s276.pdf]

Supplemental Figure 17

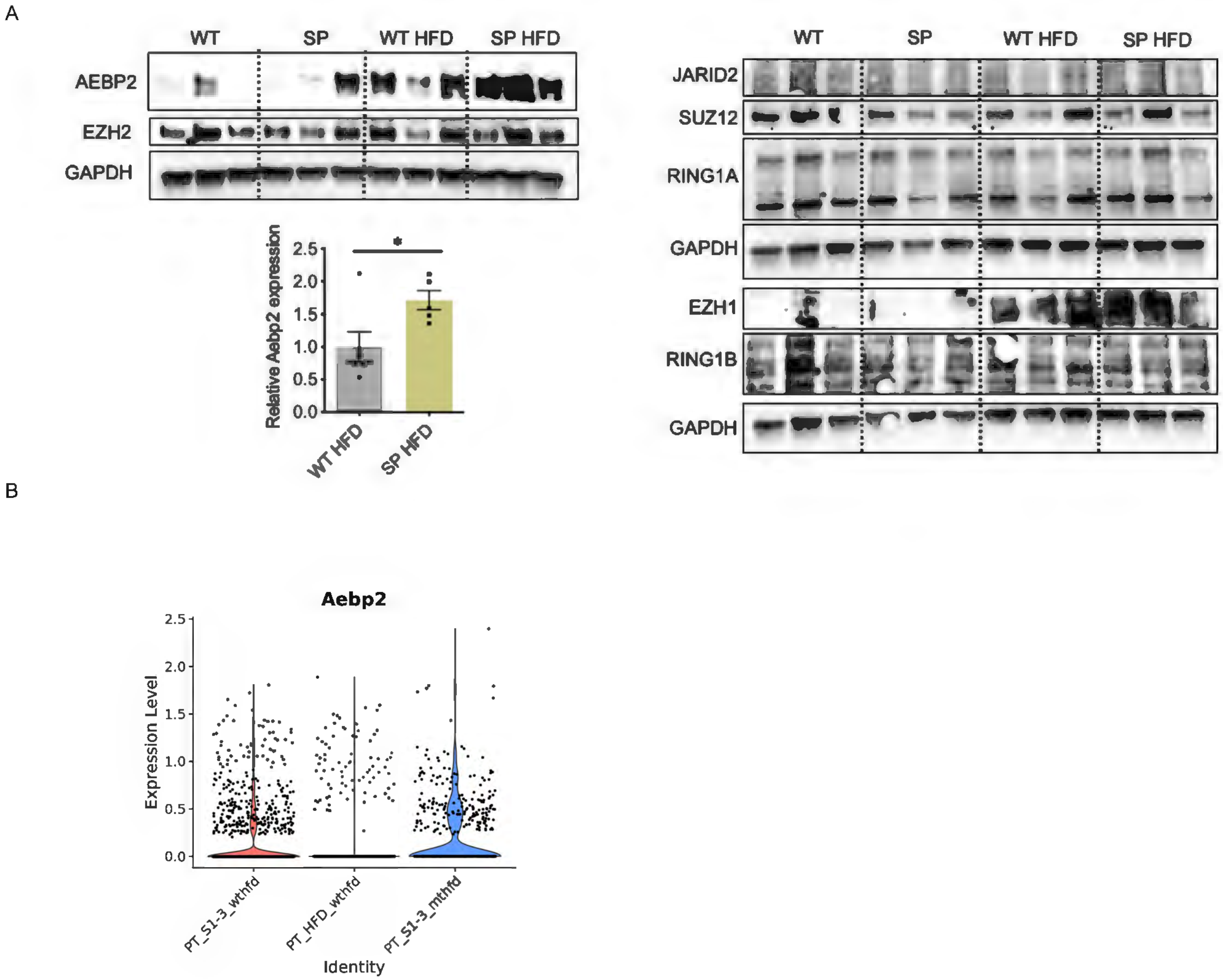

Supplemental Figure 17: Analysis of PRC2 component

(A)Western blot images of PRC2 components across experimental groups. Quantification of AEBP2 indicates increased expression in the renal cortex of SP-HFD mice compared to WT-HFD. (B) Aebp2 expression in proximal tubular cells (PTCs). Aebp2 expression is reduced in PT-HFD but elevated in the PTCs of SP-HFD mice. *$p < 0.05$, Student's t test.

Supplemental Figure 16

Related to Figure 8

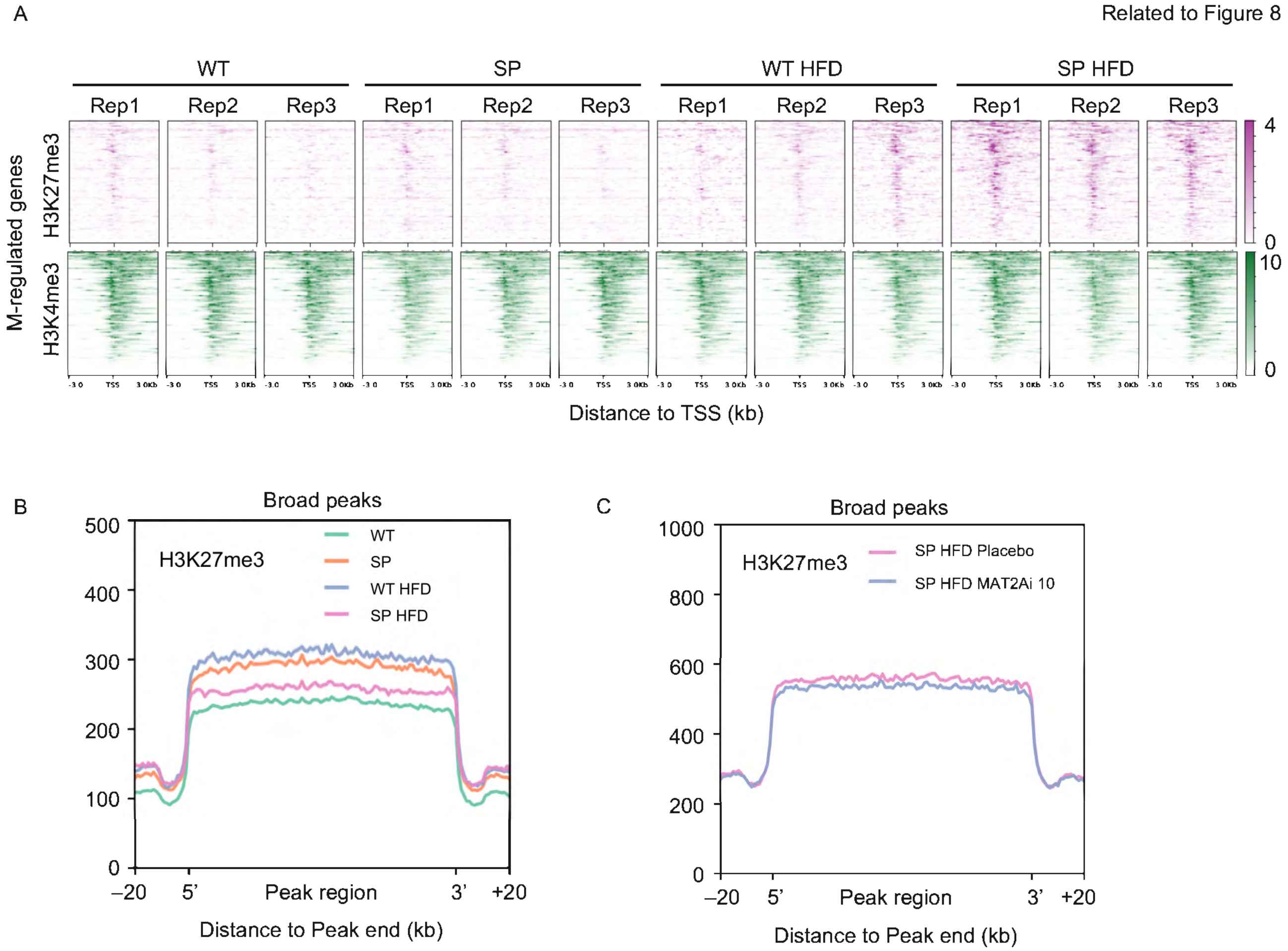

Supplemental Figure 16: Analysis of H3K27me3 and H3K4me3 CUT&RUN data.
Related to Figure 8.
(A) Heatmap showing CUT&RUN signal for H3K27me3 and H3K4me3 at the M-regulated genes. Data from three mice for each condition are shown. Signal is centered on the transcription start site (TSS). The scale of signal is shown as RRPM x 10^3. N = 90. One of the replicates was shown in Fig. 7b. (B) and (C) Metaprofiles of CUT&RUN H3K27me3 signal at the broad peak regions. The y-axis indicates the mean H3K27me3 signal (RRPM).

Supplemental Figure 15

Related to Figure 8

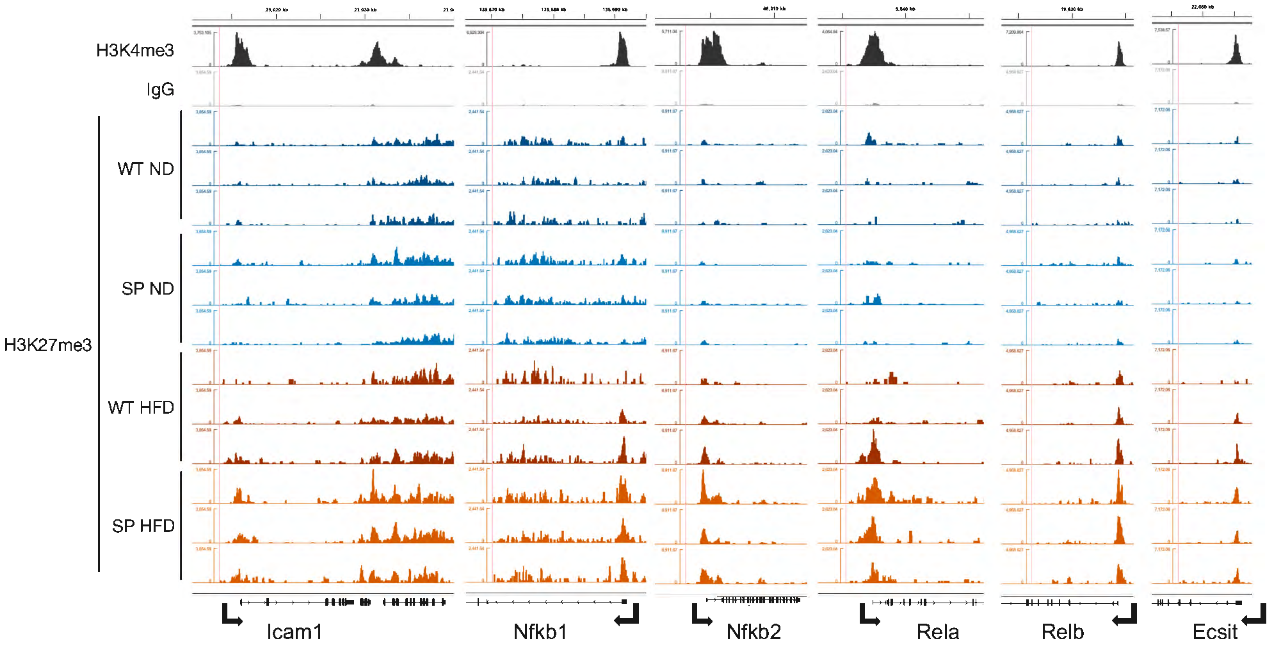

Supplemental Figure 15: Analysis of H3K27me3 CUT&RUN data profiles at NF-κB Target Genes.
Related to Figure 8.
Track for Icam1, Nfkb1, Nfkb2, Rela, Relb and Ecsit of H3K27me3 CUT&RUN in the indicated mouse renal cortex. Data from three mice for each condition are shown. The y-axis indicates reference-normalized reads per million (RRPM).

Supplemental Figure 14

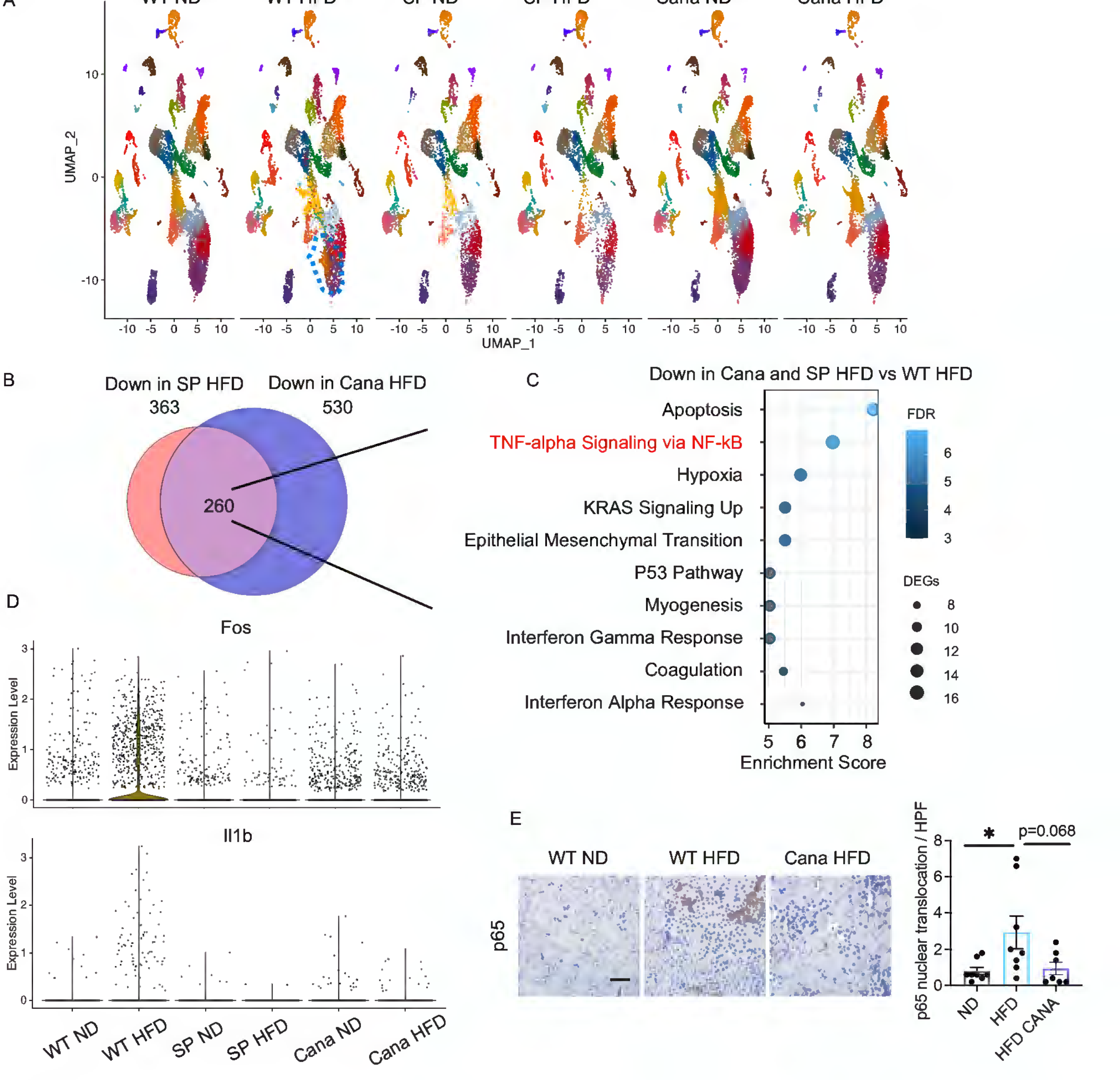

Supplemental Figure 14: Canagliflozin reduced NF-κB activation in PTCs.
(A) UMAP divided by experimental groups. PT-HFD of WT^{HFD} is highlighted with a blue dotted line. (B) Venn diagram showing genes commonly downregulated in the PTCs of SP^{HFD} and Cana-HFD compared to PTCs of WT^{HFD}. (C) Bubble plot showing pathway enrichment; commonly downregulated pathways in the PTCs of SP^{HFD} and Cana-HFD compared to PTCs of WT^{HFD} (size of the dot indicates the number of differential expression genes and color indicates FDR value). (D) Violin plot showing *Fos and Il1b* expressions in PTCs across the groups. (E) Representative images of Immunohistochemistry of p65 in the kidney and quantification (right panel). Scale Bar 50m, ND-Placebo, n=11; ND-CANA, n=11; HFD-Placebo, n=9; HFD-CANA, n=12. One-way ANOVA * P<0.05 by Tukey test.

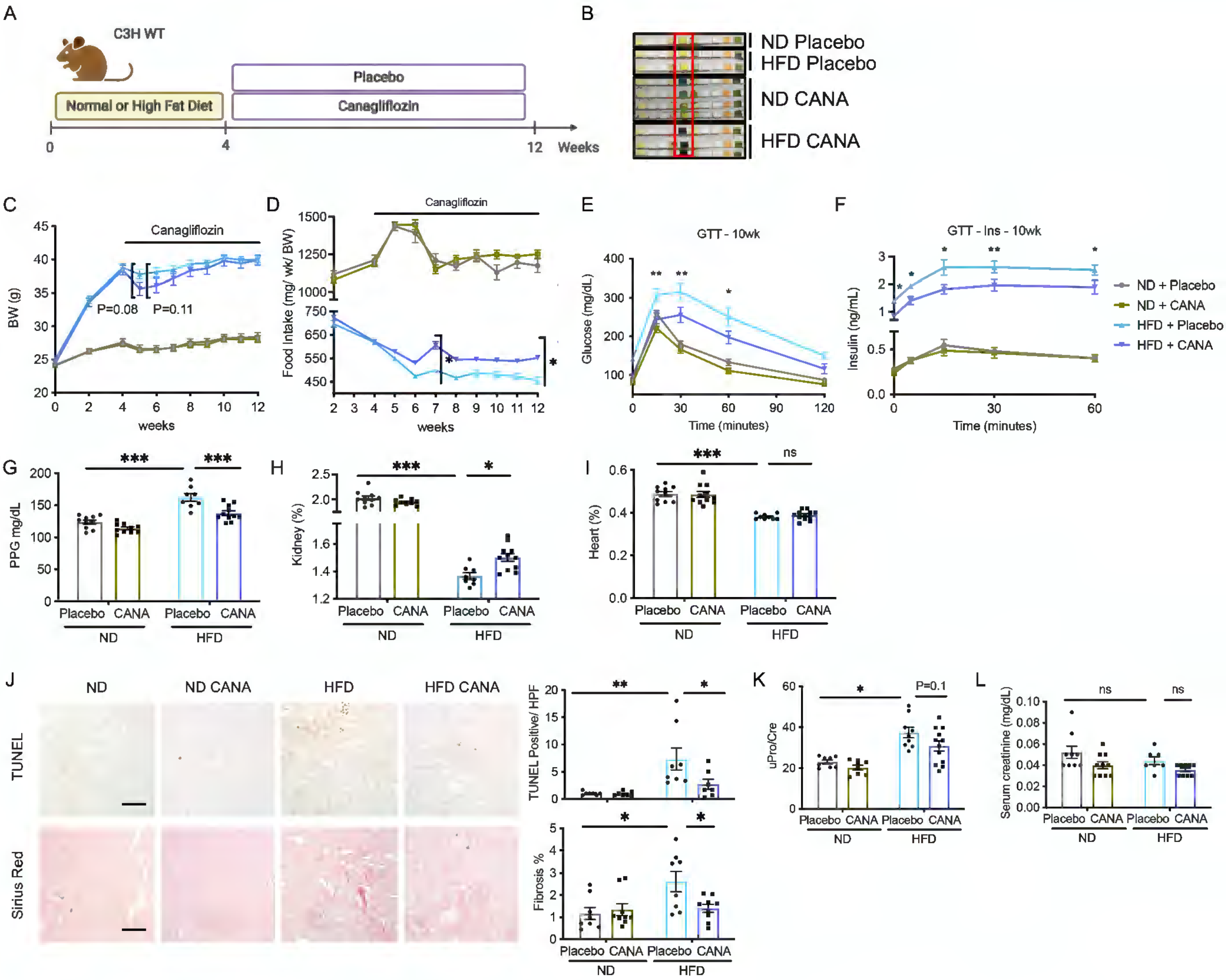

Supplemental Figure 13: Canagliflozin improved glucose intolerance and kidney injury
(A) Schematic of experimental protocol. (B) Urine strips. The red box shows portion of strip that detects glucose. Green color indicates high glucose in urine. Yellow color is negative. (C) Chronological body weight change. (D) Chronological changes in food intake. HFD elevated (E and F) glucose intolerance and insulin secretion. The elevations were improved by Cana (E and F). (G) Post-prandial blood glucose level. (H) Kidney weight, kidney weight to body weight ratio, (I) Heart weight and heart weight to body weight ratio. (J) Representative images of TUNEL staining and Sirius Red staining across groups. Right panel, quantification. (K) Urinary protein to creatinine ratio. (L) Serum creatinine level. Sample number; ND-Placebo, n=11; ND-CANA, n=11; HFD-Placebo, n=9; HFD-CANA, n=12. One-way (G-L) or Two-way ANOVA (C-F) *** P<0.001, ** P<0.01, * P<0.05 by Tukey test.

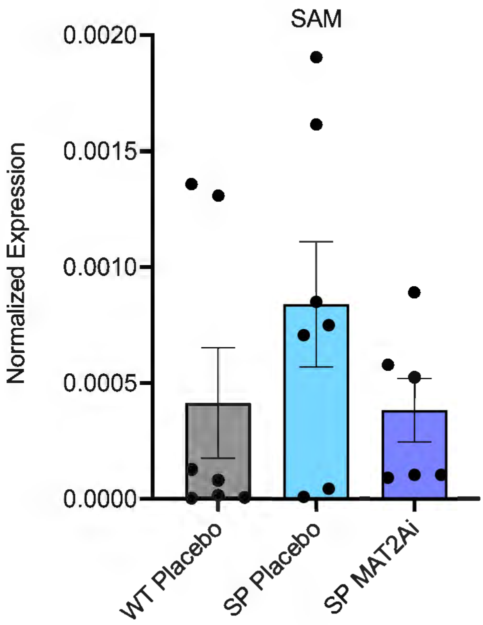

Supplemental Figure 12: Relative expression of SAM in renal cortex, WT-PL, n=7; SP-PL, n=7, SP-MAT2Ai LD, n=6.

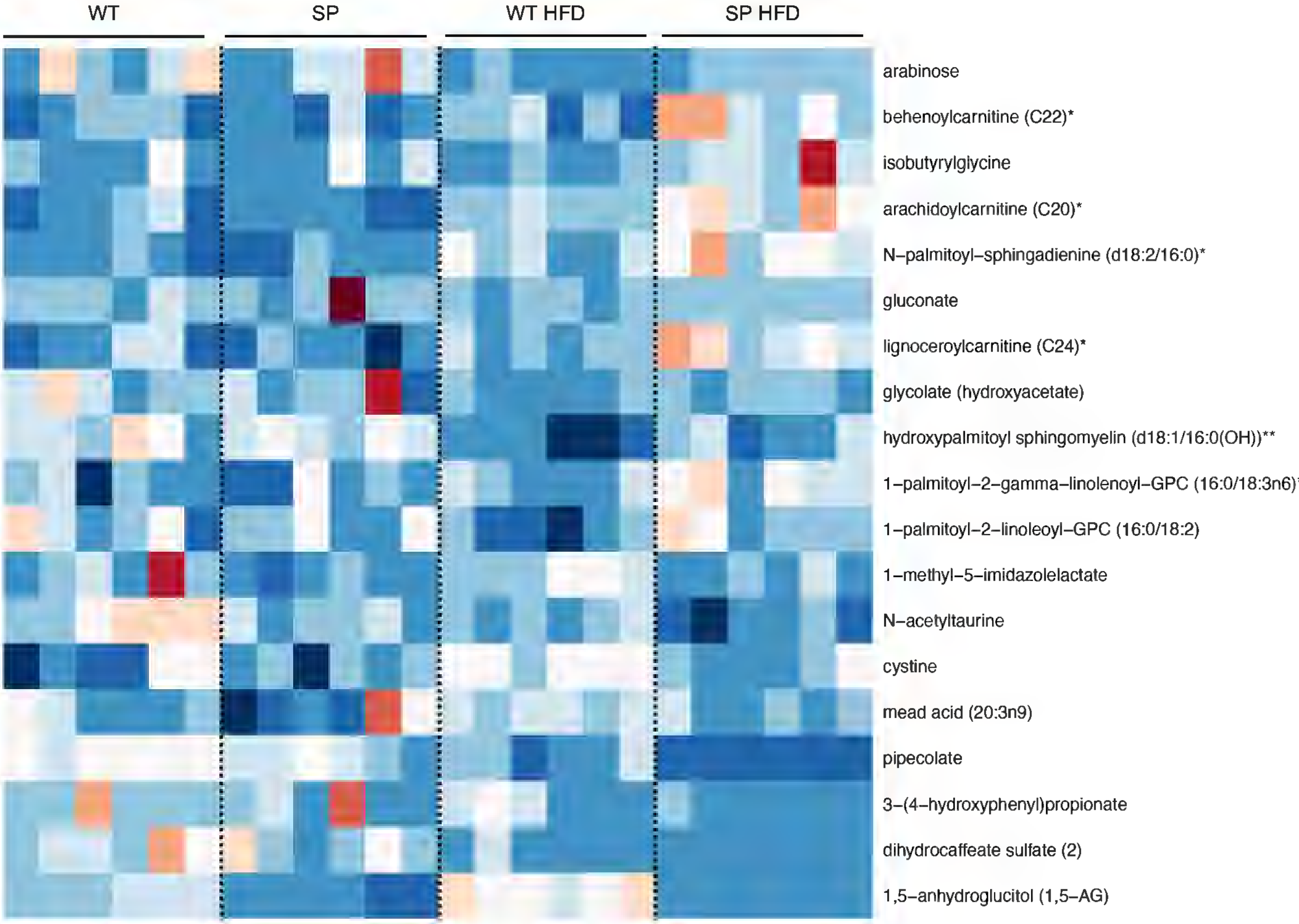

Supplemental Figure 11: Metabolomic data from serum
Related to Figure 4.
Heatmap of the untargeted metabolomics in the serum around the groups. The metabolites which are significantly different in comparison with WT-HFD and SP-HFD are shown. n=6 per group.

Supplemental Figure 10

Related to Figure 4

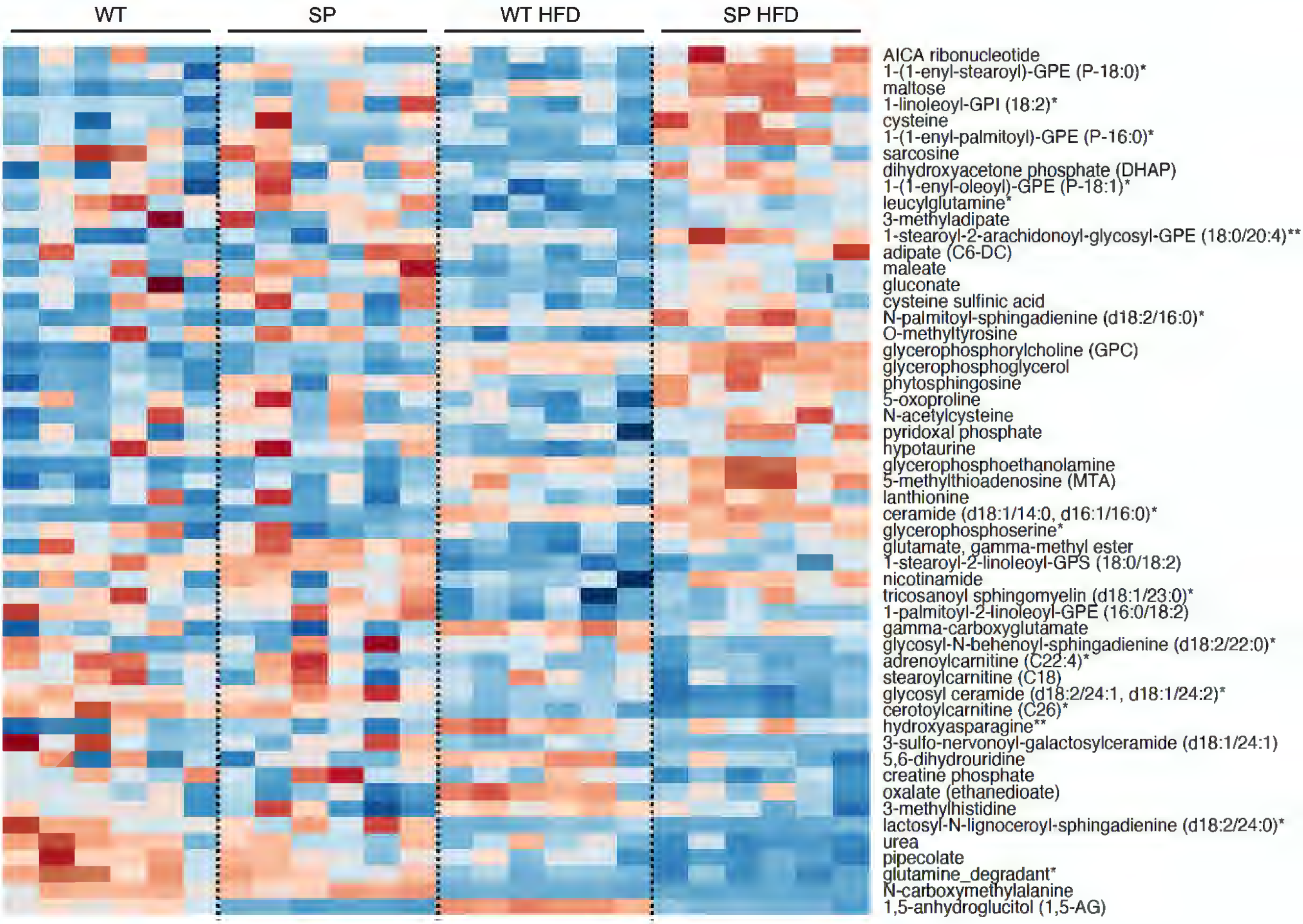

Supplemental Figure 10: Metabolomic data from renal cortex
Related to Figure 4.
Heatmap of the untargeted metabolomics in the renal cortex around the groups. The metabolites which are significantly different in comparison with WT-HFD and SP-HFD are shown. n=6 per group.

Supplemental Figure 9
Related to Figure 3 and 8

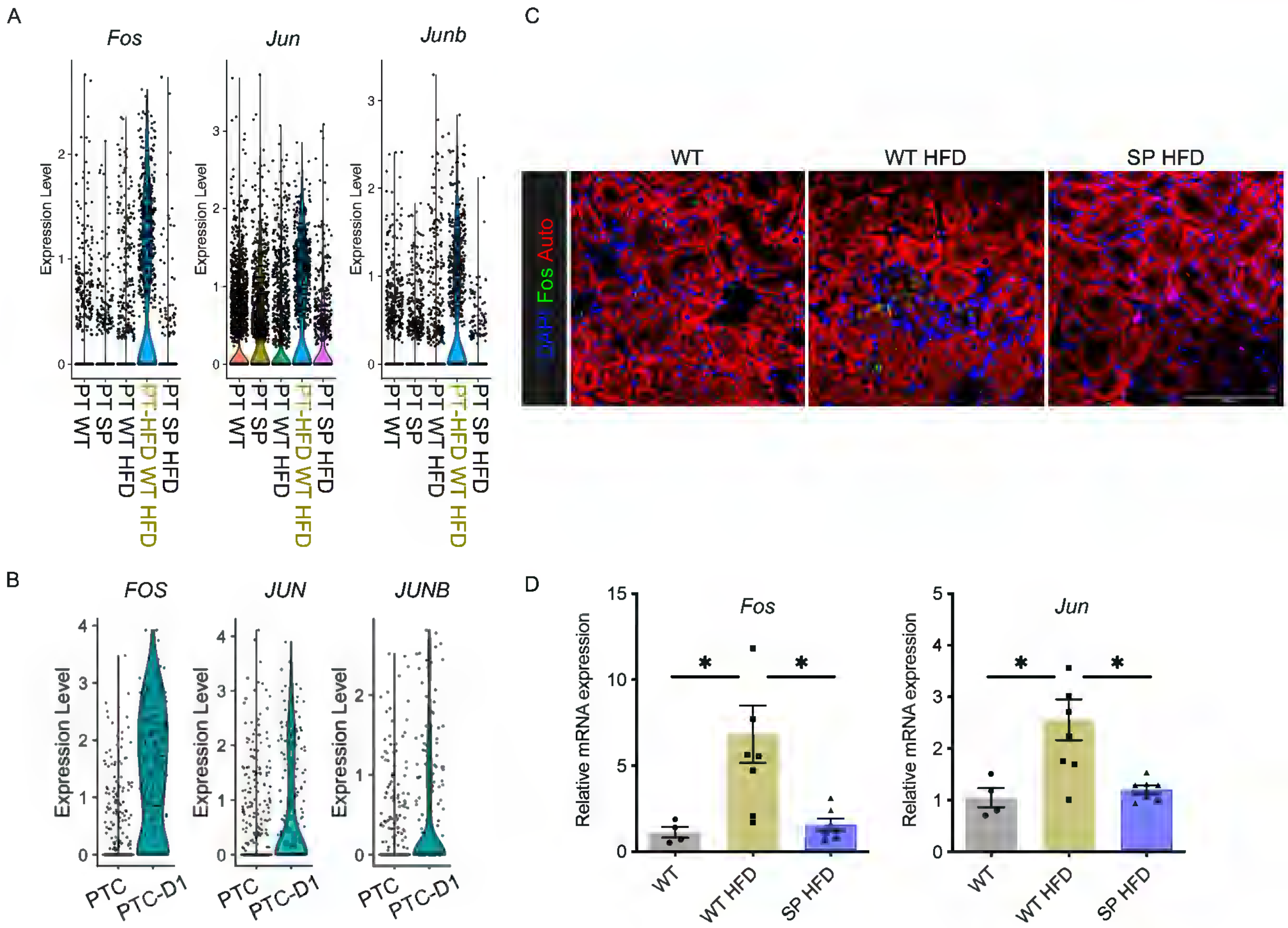

Supplemental Figure 9: AP-1 expression is upregulated in DKD
Related to Figure 3 and 8.
Violin plot showing *Fos*, *Jun* and *Junb* upregulation in mouse PT_HFD (A) and human PTC_D1 (B). (C) Representative RNAscope image showing *Fos* expression. (D) Transcript levels of *Fos* and *Jun* in the renal cortex around groups. WT, n=4; WT-HFD, n=8; HFD, n=7. One-way ANOVA, ** P<0.01, * P<0.05 by Tukey test.

Supplemental Figure 8

Related to Figure 2, 3 and 4

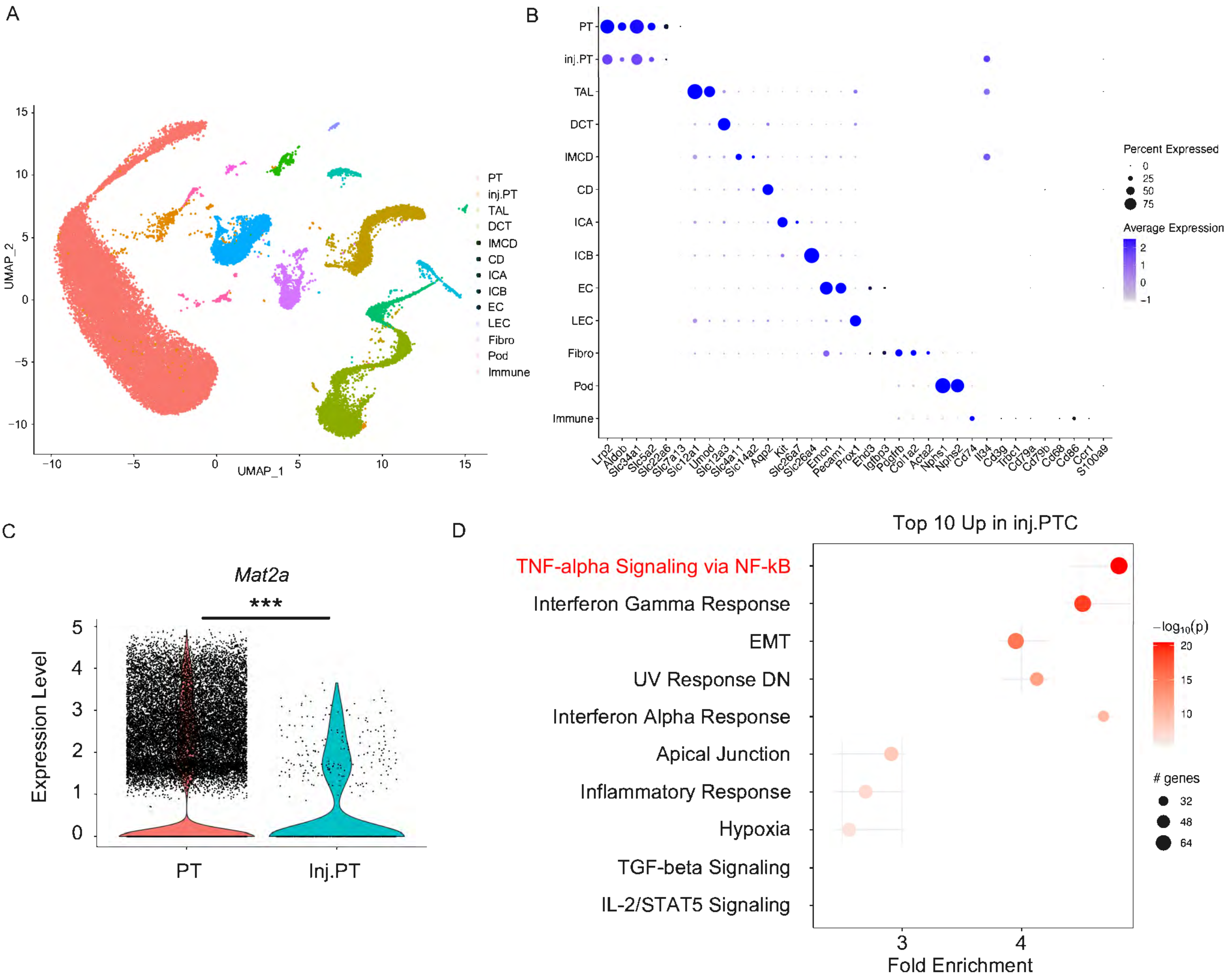

Supplemental Figure 8: Single cell RNA sequence in reported DKD model
Related to Figure 2, 3 and 4.
(A) UMAP demonstrating 13 distinct cell types in kidney. (B) Bubble plots of cellular marker genes (size of the dot indicates the % positive cells and color indicates relative expression). (C) Violin plot showing *Mat2a* expression in PTC vs inj.PTC. (D) Bubble plot showing upregulated enrichment pathways in inj.PTC (size of the dot indicates the % positive cells and color indicates relative expression). *** Adjusted P<0.001.

Supplemental Figure 7

Related to Figure 2, 3 and 4

A

Up in dPT

Term	P-value	Adjusted P-value
Oxidative Phosphorylation	5.73E-46	2.86E-44
Myc Targets V1	1.76E-39	4.39E-38
DNA Repair	1.86E-15	3.10E-14
Interferon Gamma Response	1.48E-10	1.85E-09
Interferon Alpha Response	1.94E-09	1.94E-08
Fatty Acid Metabolism	1.05E-08	8.76E-08
p53 Pathway	2.50E-07	1.79E-06
Adipogenesis	6.49E-07	4.06E-06
Apoptosis	1.37E-06	7.62E-06
Cholesterol Homeostasis	4.52E-06	2.26E-05
Androgen Response	9.85E-06	4.48E-05
Apical Junction	1.09E-04	4.55E-04
Estrogen Response Late	2.31E-04	8.90E-04
Xenobiotic Metabolism	4.75E-04	0.00155391
Glycolysis	4.75E-04	0.00155391
Coagulation	4.97E-04	0.00155391
Protein Secretion	8.77E-04	0.00249005
mTORC1 Signaling	9.46E-04	0.00249005
Allograft Rejection	9.46E-04	0.00249005
Reactive Oxygen Species Pathway	0.00130772	0.00326931
Estrogen Response Early	0.00617022	0.01469101
PI3K/AKT/mTOR Signaling	0.01304944	0.02965783
E2F Targets	0.01828509	0.03975019
Pperoxisome	0.02351096	0.04898117
UV Response Up	0.02518279	0.05036559
TNF-alpha Signaling via NF-kB	0.02993253	0.0554306
Hypoxia	0.02993253	0.0554306
Myc Targets V2	0.03780345	0.06750615
Myogenesis	0.04736055	0.07893424
Epithelial Mesenchymal Transition	0.04736055	0.07893424

B

Up in aPT

Term	P-value	Adjusted P-value
Epithelial Mesenchymal Transition	9.32E-22	4.66E-20
Interferon Gamma Response	1.97E-08	3.28E-07
KRAS Signaling Up	1.97E-08	3.28E-07
Inflammatory Response	7.07E-08	8.84E-07
TNF-alpha Signaling via NF-kB	8.12E-07	6.76E-06
Estrogen Response Late	8.12E-07	6.76E-06
Estrogen Response Early	7.92E-06	5.65E-05
Interferon Alpha Response	2.37E-05	1.48E-04
Angiogenesis	5.22E-05	2.73E-04
Apoptosis	5.46E-05	2.73E-04
Apical Junction	1.74E-04	7.57E-04
Coagulation	1.82E-04	7.57E-04
Hypoxia	4.47E-04	0.00171764
Complement	0.0011	0.0039
Myogenesis	0.0056	0.0178
UV Response Dn	0.0057	0.0178
Allograft Rejection	0.0117	0.0344
IL-6/JAK/STAT3 Signaling	0.0296	0.0823
IL-2/STAT5 Signaling	0.0419	0.1103

C

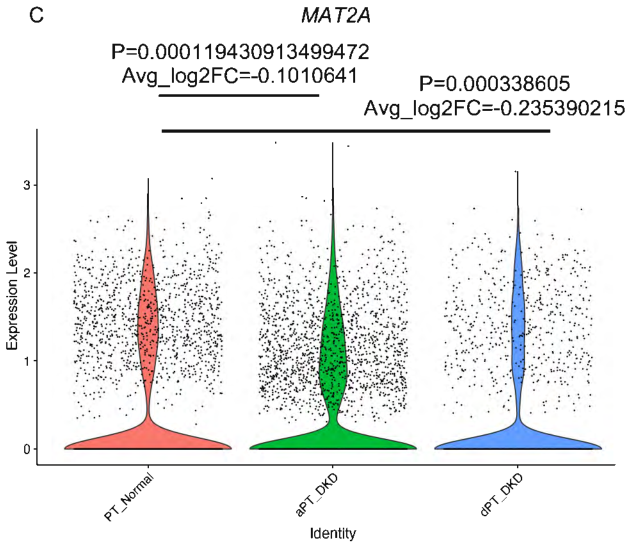

Supplemental Figure 7: Single cell RNA sequence in DKD patients of KPMP dataset.
Related to Figure 2, 3 and 4.
Pathway enrichment of dPT (A) and aPT (B). (C) Violin plot showing *MAT2A* expression in PT vs aPT and dPT.

Supplemental Figure 6

Related to Figure 2, 3 and 4

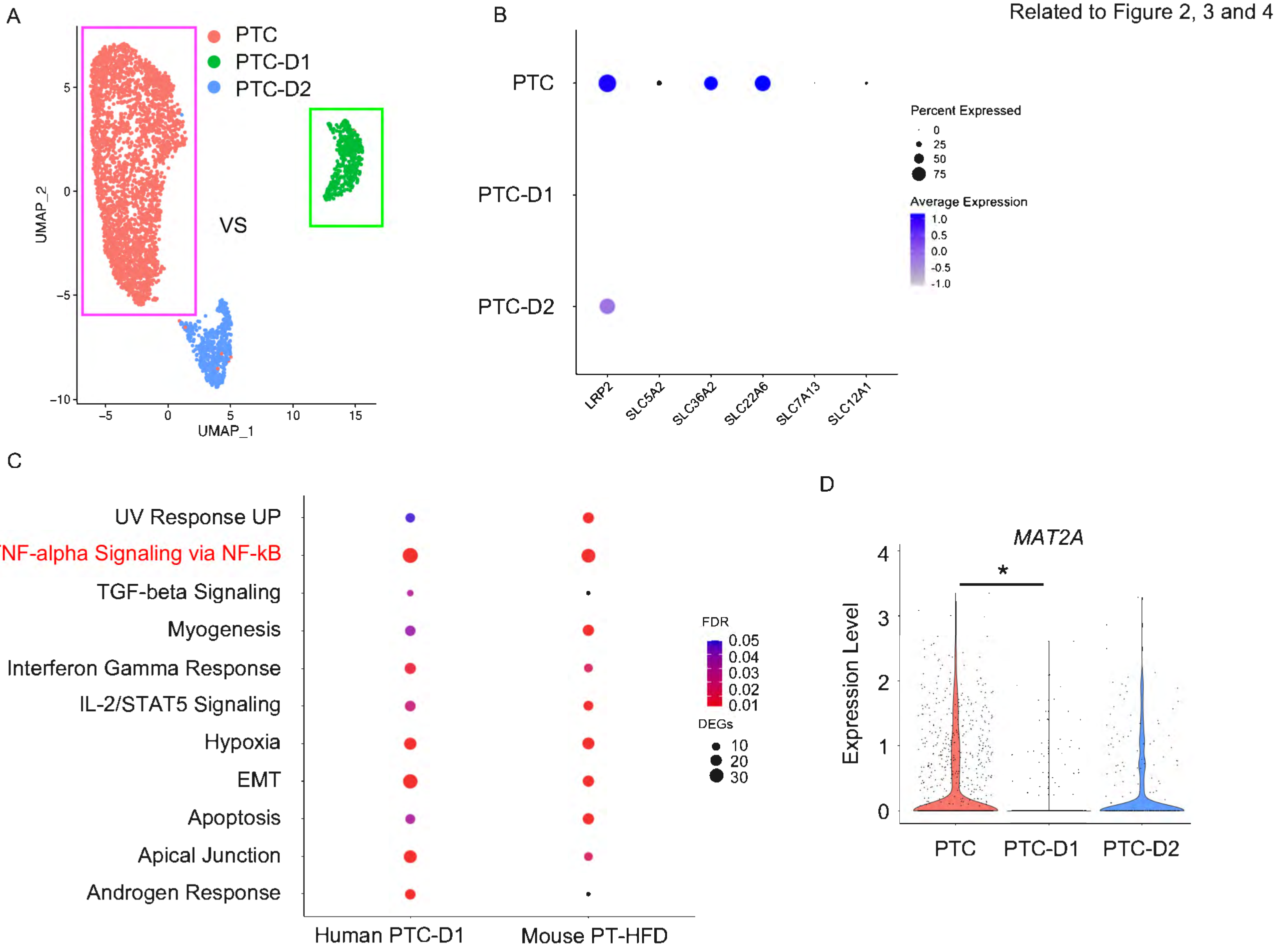

Supplemental Figure 6: Single nucleus RNA sequence in human DKD
Related to Figure 2, 3 and 4.

(A) The UMAP of 3 distinct proximal tubular cell types. (B) Bubble plots of cellular marker genes (size of the dot indicates the % positive cells and color indicates relative expression). (C) Bubble plots of pathway enrichment of human PTC-D1 and mouse PT-HFD. (D) Violin plot showing *MAT2A* expression in PTC vs PTC-D1 and D2. * Adjusted $P<0.05$.

Supplemental Figure 5
Related to Figure 3

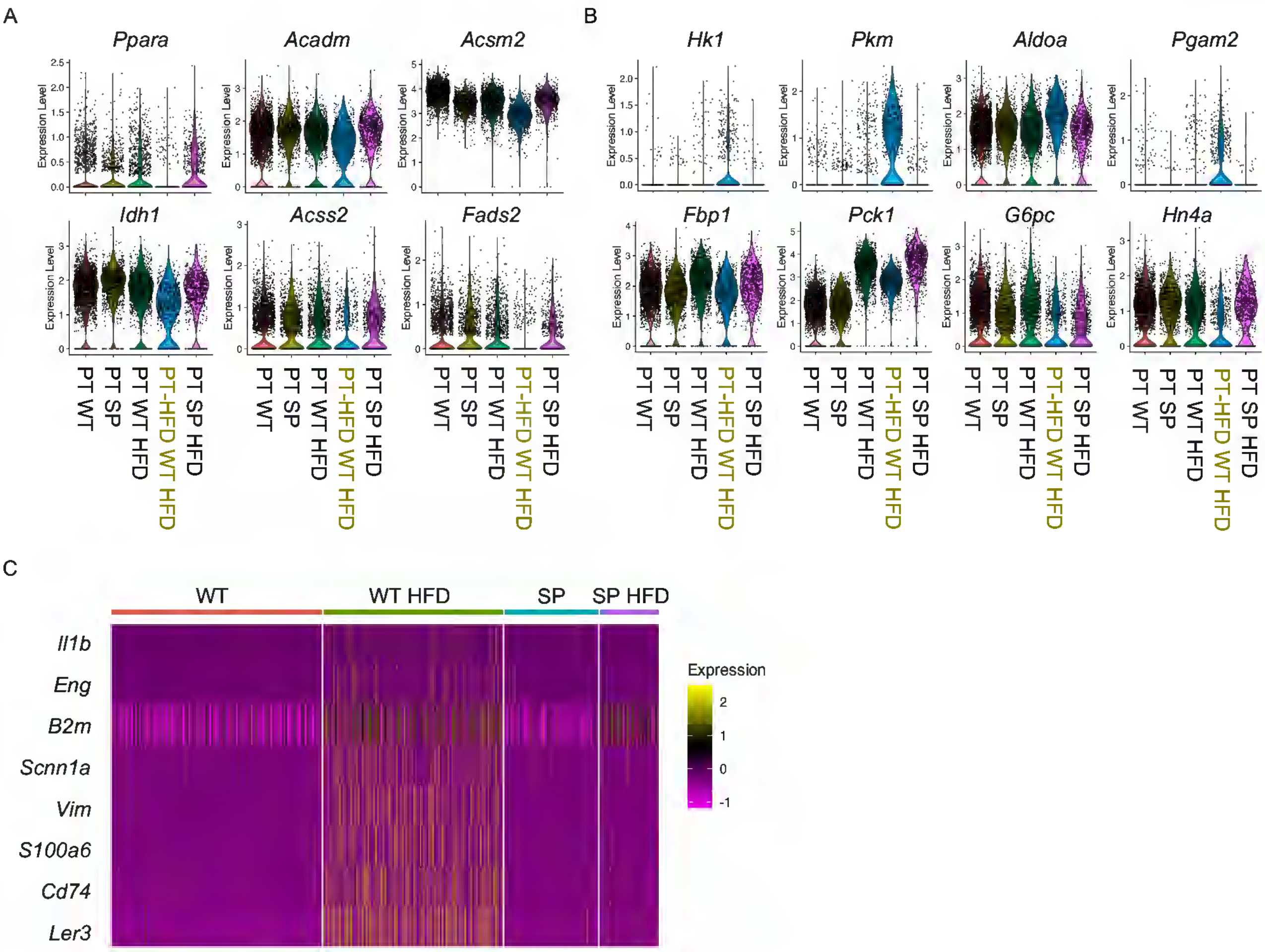

Supplemental Figure 5: Fatty acid and glucose metabolism is altered in PT-HFD
Related to Figure 3.
Representative gene expression related to (A) fatty acid oxidation and synthesis, (B) glycolysis and gluconeogenesis in PTC. (C) Heatmap of NF-κB target genes across groups.

Supplemental Figure 4

Related to Figure 2

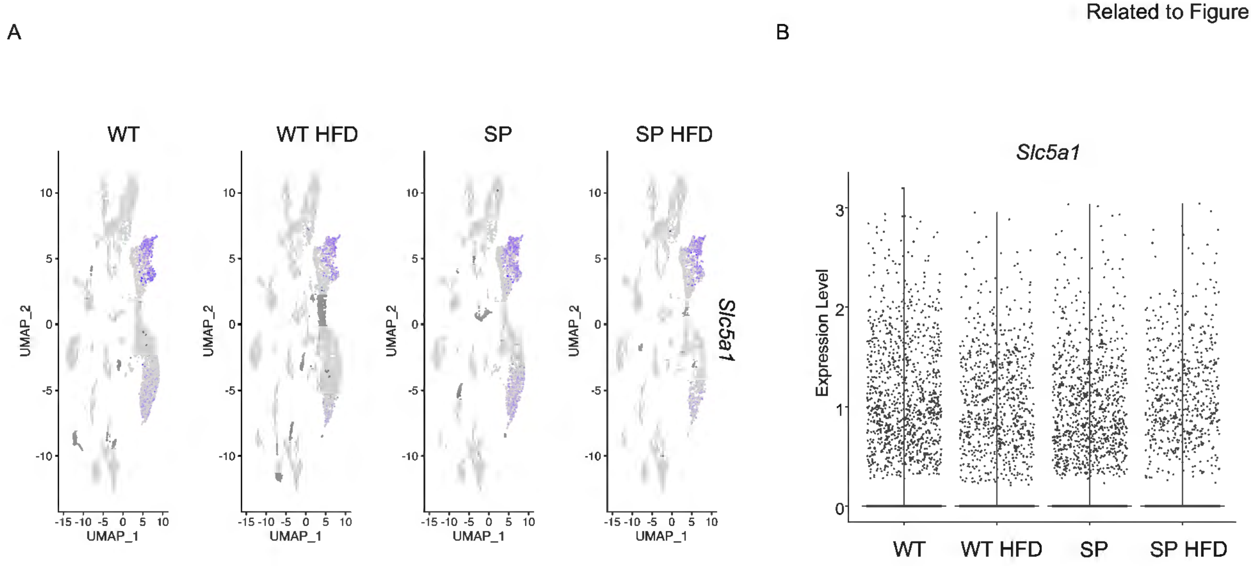

Supplemental Figure 4: SGLT1 expression across groups
Related to Figure 2.
Feature plot (A) and Violin plot (B) of *Slc5a1 gene* expression, which encodes the SGLT1 co-transporter.

Supplemental Figure 3

Related to Figure 2

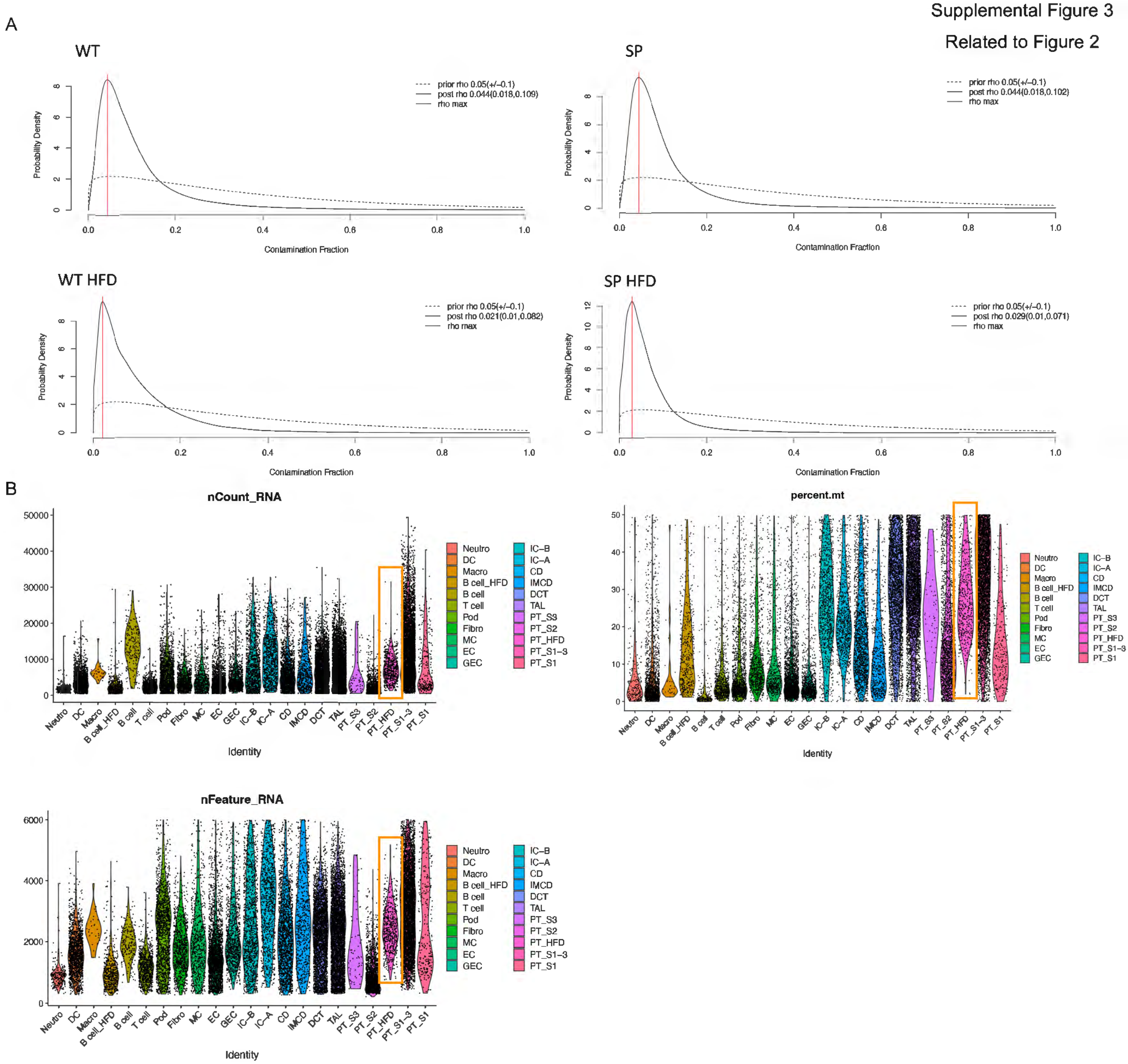

Supplemental Figure 3: Quality control metrics of scRNA-seq
Related to Figure 2.
(A) Distribution of ambient RNA contamination fraction (rho) before and after SoupX correction. (B) Quality control metrics (nCount_RNA, percent.mt, and nFeature_RNA) across cell clusters. PT-HFD is highlighted with an orange box.

Supplemental Figure 2

Related to Figure 2

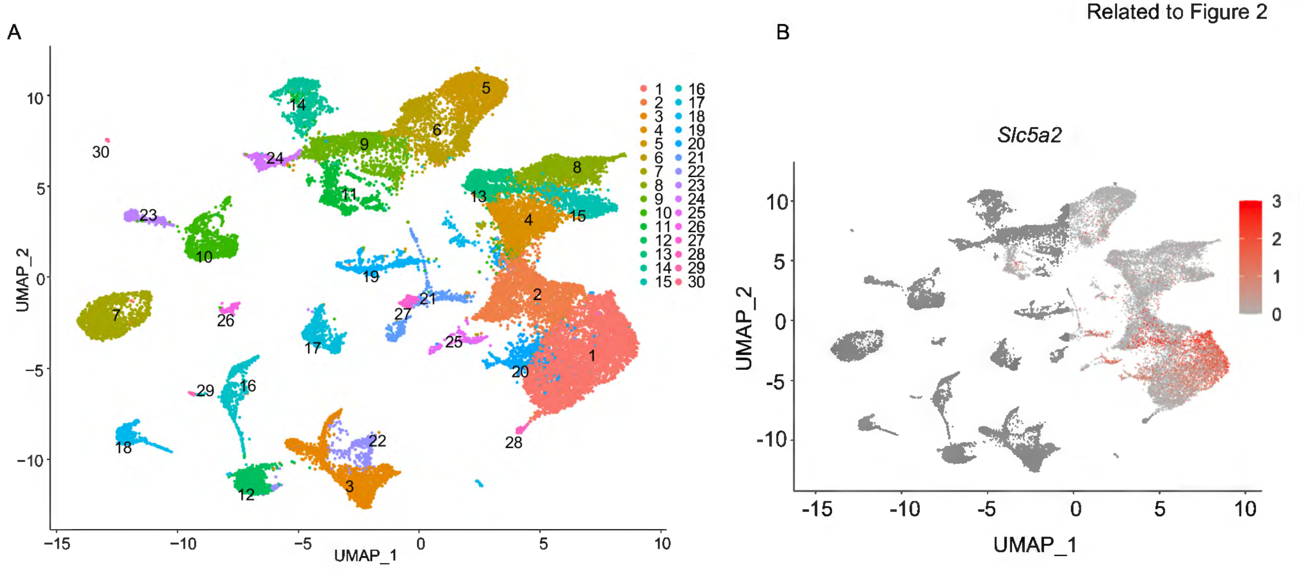

Supplemental Figure 2: SGLT2 expression across groups

Related to Figure 2.

(A) UMAP of 30 distinct cell types. (B) Feature plot of SGLT2.

Supplemental Figure1

Related to Figure 1 and 3

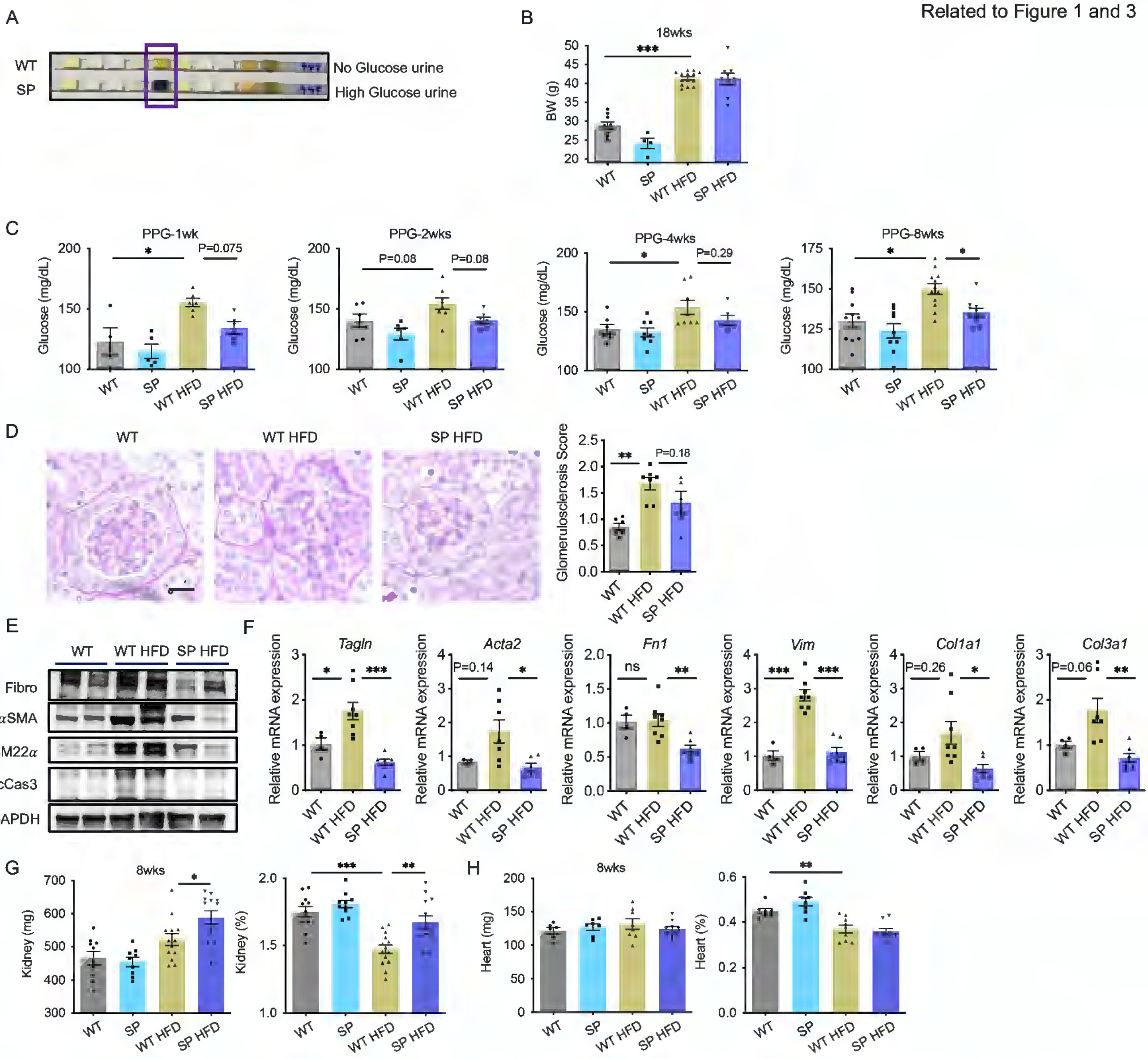

Supplemental Figure 1: Urine test, BW, glomerular injury scoring, fibrotic changes, kidney and heart size in mice at 8 or 18 week-feeding timepoint

Related to Figure 1 and 3.

(A) Urine strips. The purple box shows portion of strip that detects glucose. Green color indicates high glucose in urine. Yellow color is negative. (B) Body weight at 18 week-feeding timepoint. (C) Post-prandial blood glucose level at 1, 2, 4 and 8 week-feeding timepoint. (D) Representative image of Glomeruli across the group, Right panel, quantification. (E) Western blot analysis of tissue harvested from renal cortex across groups. (F) RT-PCR analysis of fibrotic genes in tissue isolated from the renal cortex across groups (G) Kidney weight, kidney weight to body weight ratio, (H) Heart weight and heart weight to body weight ratio at 8 week-feeding timepoint. Scale Bar, 100μm Sample number; WT-ND 8wks, n=10; SP-ND 8wks, n=9; WT-HFD 8wks, n=14; SP-HFD 8wks, n=13; WT-ND 18wks, n=9; SP-ND 18wks, n=4; WT-HFD 18wks, n=13; SP-HFD 18wks, n=9. One-way ANOVA *** P<0.001, ** P<0.01, * P<0.05 by Tukey test. The GAPDH blot shown here is the same as that in Figure 3B

Supplemental Table 4

Primer sequence

Primer: mouse HPRT1 Forward: CAGTCCCAGCGTCGTGATTA
Primer: mouse HPRT1 Reverse: TGGCCTCCCATCTCCTTCAT
Primer: mouse ICAM1 Forward: TGGTCACCGTTGTGATCCCT
Primer: mouse ICAM1 Reverse: CTCCACACTCTCCGGAAACGAATA
Primer: mouse IL-6 Forward: TCCAGTTGCCTTCTTGGGAC
Primer: mouse IL-6 Reverse: GTGTAATTAAGCCTCCGACTTG
Primer: mouse TNF Forward: CCCTCACACTCAGATCATCTTCT
Primer: mouse TNF Reverse: GCTACGACGTGGGCTACAG
Primer: mouse CCL2 Forward: CAGCTCTCTCTTCCTCCACCAC
Primer: mouse CCL2 Reverse: GCGTTAACTGCATCTGGCTGAG
Primer: mouse IL-1**β** Forward AGAAGAGCCCATCCTCTGTGACTCATGG
Primer: mouse IL-1**β** Reverse CACACACCAGCAGGTTATCATCA
Primer: mouse Havcr1 Forward TCAGGAAGCTGAGCAAACATTA
Primer: mouse Havcr1 Reverse AGTGCCATTCCAGTCTGGTT
Primer: mouse Fn1 Forward ATGAGAAGCCTGGATCCCCT
Primer: mouse Fn1 Reverse GGAAGGGTAACCAGTTGGGG
Primer: mouse Tagln Forward CGGCCTTTAAACCCCTCACC
Primer: mouse Tagln Reverse CATGTTGAGGCAGAGAAGGCT
Primer: mouse ACTA2 Forward AGCCATCTTTCATTGGGATGG
Primer: mouse ACTA2 Reverse CCCCTGACAGGACGTTGTTA
Primer: mouse mCol3a1 Forward TGACTGTCCCACGTAAGCAC
Primer: mouse mCol3a1 Reverse GAGGGCCATAGCTGAACTGA
Primer: mouse Forward CGATGGATTCCCGTTCGAGT
Primer: mouse Reverse CGATCTCGTTGGATCCCTGG
Primer: human IL-6 Forward: GGTACATCCTCGACGGCATCT
Primer: human IL-6 Reverse: GTGCCTCTTTGCTGCTTTCAC
Primer: human IL-8 Forward: AAGGAAAACTGGGTGCAGAG
Primer: human IL-8 Reverse: ATTGCATCTGGCAACCCTAC
Primer: human TNF Forward: CCCTCACACTCAGATCATCTTCT
Primer: human TNF Reverse: GCTACGACGTGGGCTACAG
Primer: human CCL20 Forward: GGCGAATCAGAAGCAAGCAA
Primer: human CCL20 Reverse: GGATTTGCGCACACAGACAA

Primer: human Fibronectin Forward: CCGCCGAATGTAGGACAAGA
Primer: human Fibronectin Forward: TGTCAGAGTGGCACTGGTAG
Primer: human GAPDH Forward: CCTCAACGACCACTTTGTCA
Primer: human GAPDH Reverse: TTACTCCTTGGAGGCCATGT

Supplemental Table 5

Antibody list

Antibody	Supplier	Catalog number
Anti-Phospho-NF-κB p65	Cell Signaling	Cat#3033
NF-κB p65 (C22B4) Rabbit monoclonal Antibody	Cell Signaling	Cat# 4764
Anti-TAGLN/Transgelin antibody	Abcam	Cat# ab14106
SGLT2 Antibody	GeneTex	Cat#GTX59872
Anti-Fibronectin antibody	Sigma	Cat# F3648
Anti-Actin, α-Smooth Muscle - Cy3™ antibody	Sigma	Cat# C6198
Cleaved Caspase-3 (Asp175) (5A1E) Rabbit mAb antibody	Cell Signaling	Cat#9664
Mouse TIM-1/KIM-1/HAVCR Antibody	R and D Systems	Cat#AF1817
Rabbit Anti-GAPDH Monoclonal Antibody, Unconjugated, Clone 14C10	Cell Signaling	Cat#2118
Donkey anti-Rabbit IgG (H+L) Highly Cross-Adsorbed Secondary Antibody, Alexa Fluor 594	Thermo Fisher Scientific	Cat# A-21207
DAPI	Sigma	Cat# D9564
Recombinant Anti-Histone H3 (tri methyl K9) antibody [EPR16601] - ChIP Grade (ab176916)	Abcam	ab176916
Tri-Methyl-Histone H3 (Lys27) (C36B11) Rabbit mAb	Cell Signaling	Cat# 9733
Tri-Methyl-Histone H3 (Lys4) (C42D8) Rabbit mAb	Cell Signaling	Cat# 9751
Rabbit (DA1E) mAb IgG XP® Isotype Control (CUT&RUN)	Cell Signaling	Cat#66362
Polycomb Group 2 (PRC2) Antibody Sampler Kit	Cell Signaling	Cat#62083
