# Supplementary material for: SGLT2 inhibition protects kidney function by SAM-dependent epigenetic repression of inflammatory genes under metabolic stress
Source: J Clin Invest. 2025 Oct 1;135(19):e188933. doi: 10.1172/JCI188933 (PMC12483609; doi:10.1172/JCI188933)

Unprocessed scans of western blot analysis.

Related to Figure 1

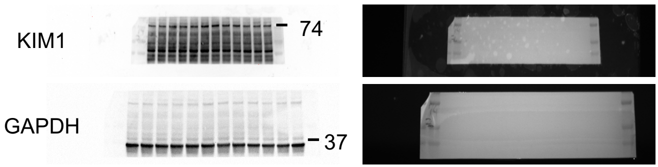

Related to Figure 3

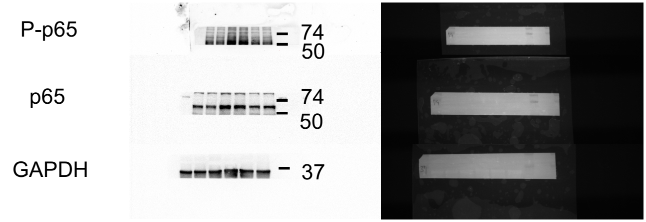

Related to Figure 7

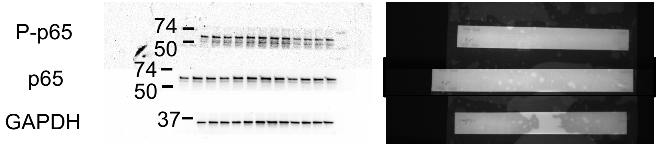

Related to Supplemental Figure 1

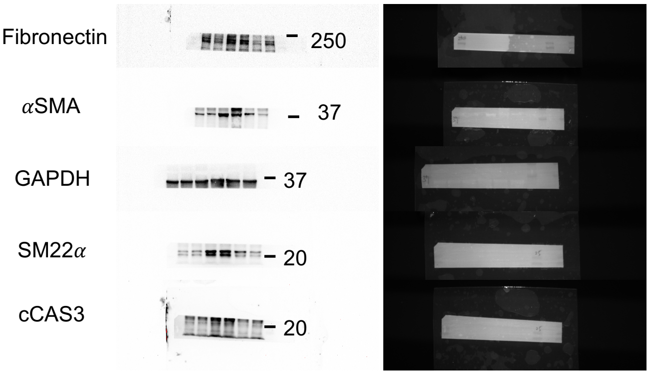

Related to Supplemental Figure 17

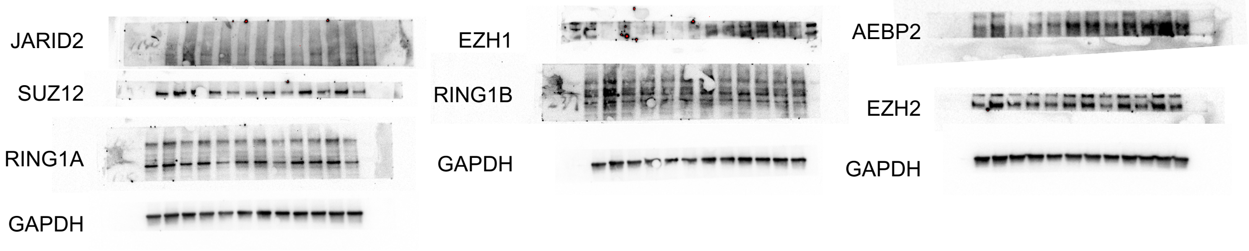

Supplement: Unedited blot and gel images [file jci-135-188933-s277.pdf]
